# Expression Quantitative Trait Loci for Extreme Host Response to Influenza A in Pre-Collaborative Cross Mice

**DOI:** 10.1534/g3.111.001800

**Published:** 2012-02-01

**Authors:** Daniel Bottomly, Martin T. Ferris, Lauri D. Aicher, Elizabeth Rosenzweig, Alan Whitmore, David L. Aylor, Bart L. Haagmans, Lisa E. Gralinski, Birgit G. Bradel-Tretheway, Janine T. Bryan, David W. Threadgill, Fernando Pardo-Manuel de Villena, Ralph S. Baric, Michael G. Katze, Mark Heise, Shannon K. McWeeney

**Affiliations:** * Pacific Northwest Regional Center of Excellence for Biodefense and Emerging Infectious Diseases Research, Portland, Oregon 97006; † Oregon Clinical and Translational Research Institute, Oregon Health & Science University, Portland, Oregon 97239; ‡ Department of Genetics, University of North Carolina, Chapel Hill, North Carolina 27599; § Department of Microbiology, School of Medicine, University of Washington, Seattle, Washington, 98195; ** Lineberger Comprehensive Cancer Center, University of North Carolina, Chapel Hill, North Carolina, 27514; †† Erasmus Medical Center, 3000 CA Rotterdam, the Netherlands; ‡‡ Department of Epidemiology, University of North Carolina, Chapel Hill, North Carolina, 27599; §§ Department of Genetics, North Carolina State University, Raleigh, North Carolina, 27695; *** Department of Microbiology and Immunology, University of North Carolina, Chapel Hill, North Carolina 27599; ††† Division of Bioinformatics and Computational Biology, Medical Informatics and Clinical Epidemiology, Oregon Health & Science University, Portland, Oregon 97239; ‡‡‡ Division of Biostatistics, Public Health & Preventative Medicine, Oregon Health & Science University, Portland, Oregon 97239; §§§ OHSU Knight Cancer Institute, Oregon Health & Science University, Portland, Oregon 97239

**Keywords:** eQTL, influenza, collaborative cross, host response, SEM, Mouse Collaborative Cross, Mouse Genetic Resource

## Abstract

Outbreaks of influenza occur on a yearly basis, causing a wide range of symptoms across the human population. Although evidence exists that the host response to influenza infection is influenced by genetic differences in the host, this has not been studied in a system with genetic diversity mirroring that of the human population. Here we used mice from 44 influenza-infected pre-Collaborative Cross lines determined to have extreme phenotypes with regard to the host response to influenza A virus infection. Global transcriptome profiling identified 2671 transcripts that were significantly differentially expressed between mice that showed a severe (“high”) and mild (“low”) response to infection. Expression quantitative trait loci mapping was performed on those transcripts that were differentially expressed because of differences in host response phenotype to identify putative regulatory regions potentially controlling their expression. Twenty-one significant expression quantitative trait loci were identified, which allowed direct examination of genes associated with regulation of host response to infection. To perform initial validation of our findings, quantitative polymerase chain reaction was performed in the infected founder strains, and we were able to confirm or partially confirm more than 70% of those tested. In addition, we explored putative causal and reactive (downstream) relationships between the significantly regulated genes and others in the high or low response groups using structural equation modeling. By using systems approaches and a genetically diverse population, we were able to develop a novel framework for identifying the underlying biological subnetworks under host genetic control during influenza virus infection.

Given the wide variation in infectious disease severity across human populations, identifying host genetic contributions to the infectious disease response is increasingly becoming an important avenue of research in infectious diseases (Aouizerat *et al.* 2011; Newport and Finan 2011; Petrovski *et al.* 2011; Rauch *et al.* 2010). Influenza A virus, the cause of annual flu outbreaks with significant morbidity and mortality, shows a wide degree of disease phenotype variation across human populations (Li *et al.* 2009; Mallia and Johnston 2007; To *et al.* 2010; Tsalik *et al.* 2010; Yu *et al.* 2008), including clear differences in transcriptional profiles after infection (Zaas *et al.* 2009). Although it has been difficult to disentangle the contributions of host genetics and environmental cofactors, there is evidence that host genetics do play some role in determining outcomes (susceptibility/resistance) of influenza infection (Albright *et al.* 2008). The genes most differentially expressed in the lung between mild and severe responders to influenza virus should provide insight into the processes and pathways that contribute to susceptibility and resistance. Gene networks that are under host genetic control are of particular interest because they help us to better understand the role host genetics plays in determining disease outcomes.

A common animal model in influenza studies is the single gene knockout mouse (Dawson *et al.* 2000; Imai *et al.* 2008; Karupiah *et al.* 1998; Kopf *et al.* 2002; Koyama *et al.* 2007; Szretter *et al.* 2007). In these studies, authors have been able to demonstrate the importance of isolated genes to infection models, but they did not consider the effect of genetic backgrounds on host response. Alternatively, in several studies authors have used panels of mouse inbred strains, including the BXD recombinant inbred panel, to examine the effect of complex genetic architecture on the response to influenza infection (Asanuma *et al.* 2001; Bender *et al.* 1995; Boon *et al.* 2009; Ding *et al.* 2008; Srivastava *et al.* 2009). These studies reported that host genetic differences do influence the transcriptional response to infection (Boon *et al.* 2009), although it was less clear how host genetic differences impacted the transcriptional environments that lead to different disease outcomes.

We investigated the genetic architecture of the host transcriptional response to Influenza A using the Collaborative Cross (CC). The CC is a panel of multiparental recombinant inbred strains with high and uniform levels of genetic variation than other currently available animal models (Collaborative Cross Consortium 2012). Recently, investigators have used mice from incipient lines of the CC that are not fully inbred (pre-CC) to demonstrate that, as expected, the CC has more extensive phenotypic diversity than standard inbred strains panels for a diversity of biomedical traits (Aylor *et al.* 2011; Durrant *et al.* 2011; Kelada *et al.* 2012; Philip *et al.* 2011). Using a very focused design to select genes that are most relevant to the host response, we identified genes that were differentially expressed between severe and mild responders to influenza infection (as determined by extent of viral replication and weight loss). We used these mice for expression quantitative trait loci, or eQTL mapping, to identify genetic polymorphisms controlling the expression of these host response relevant transcripts. Validation of the significant eQTL was conducted by measuring the expression levels in the progenitor strains (Wayne and Mcintyre 2002) and relating those measurements to the allele effects predicted using the pre-CC. Finally, structural equation modeling (SEM) allowed us to identify additional genes that had putative causal or downstream relationships with the validated eQTL candidate genes, using an adaptation of previous methods for simpler crosses (Aten *et al.* 2008). This approach allowed us to use a high confidence network of gene−marker relationships to drive discovery of other genes important in the host response that are differentially expressed while only being indirectly regulated by specific loci.

## Materials and Methods

### Animals

Female mice (8−16 weeks of age) from the eight founder strains (A/J, C57BL/6J, 129S1/SvImJ, NOD/ShiLtJ, NZO/HILtJ, CAST/EiJ, PWK/PhJ, and WSB/EiJ) were originally obtained from The Jackson Laboratory (http://www.jax.org) but bred at UNC-Chapel Hill under specific pathogen free conditions. Pre-CC lines used in this study are part of the U.S. arm of the CC project (Collaborative Cross Consortium 2012). Mice were bred at Oak Ridge National Laboratories under specific pathogen free conditions (Threadgill *et al.* 2011), shipped to North Carolina and transferred directly into a BSL-3 containment laboratory at UNC-Chapel Hill. All experiments were approved by the UNC Institutional Animal Care and Use Committee (IACUC protocol number 08-142).

### Virus and cell lines

The mouse adapted influenza A strain A/PR/8/34 (H1N1) was used for all infection studies. A/PR/8/34 stocks were made by infection of 10-day-old embryonated chicken eggs. MDCK cells grown in high glucose Dulbecco's minimal eagle's medium (10% fetal bovine serum, 1% penicillin/streptomycin) were used for titering virus by plaque assay.

### Infections

Mice were lightly anesthetized via inhalation with Attane Isoflurane (Minrad Inc, Orchard Park, NY). After they were anesthetized, mice were infected intranasally with 5 × 10^−2^ pfu of PR8 in 50 μL of phosphate-buffered saline. Mice were assayed daily for morbidity (determined as % weight loss), mortality, and clinical disease scores. At 4 days after infection, mice were killed via an overdose of isoflurane and tissues were taken for various assays.

### Immunohistochemical (IHC) analysis of viral replication

For detection of influenza virus antigen, we used serial sections from formalin-fixed, paraffin-embedded lung samples. After deparaffinization and rehydration, antigen retrieval was performed using 0.1% protease (10 min at 37°). Endogenous peroxidase was blocked with 3% hydrogen peroxide, and slides were briefly washed with phosphate-buffered saline/0.05% Tween 20. Mouse anti-influenza virus nucleoprotein (clone Hb65; ATCC) and horseradish peroxidase−labeled goat antimouse IgG2a were used for 1 hr at room temperature. Peroxidase activity was revealed by incubating slides in 3-amino-9-ethylcarbazole (Sigma-Aldrich) for 10 min, resulting in a bright-red precipitate, followed by counterstaining with hematoxylin. Tissue sections from noninfected BALB/c mice and mouse IgG2a isotype antibody (R&D) were used as negative controls. The extent of influenza viral antigen spread across these slides was then scored in a blinded fashion on a 0-5 scale.

### Selection of mouse lines

**Figure 1:**
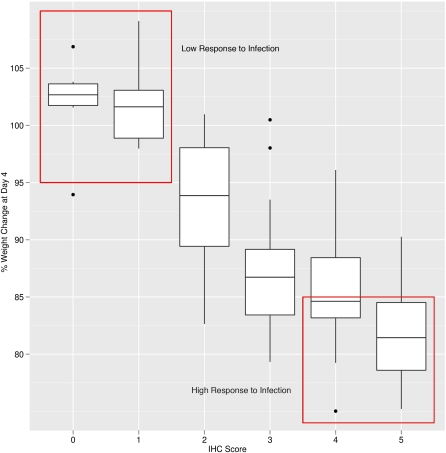
Phenotypic distribution of pre-CC lines used for categorization Two groups (red boxes) correspond to mice that displayed HRI or LRI. The y-axis displays the percent change in weight at 4 DPI, relative to a baseline. The x-axis displays an IHC score (see *Materials and Methods*) that was an indicator of infection severity.

From an initial set of 99 female mice, derived from 99 CC lines we selected mice at both extremes of the host response distribution (Figure 1; supporting information, Figure S1) for transcriptional profiling. Mice were selected to be part of the low response to infection (LRI) group if they had weight change at day 4 postinfection (4 DPI) of <5% and an IHC score of either 0 or 1 (18 samples). Mice were selected for the high response to infection (HRI) group if they had weight change of >15% and an IHC score of either 4 or 5 (26 samples).

### Genotyping

We genotyped each of the infected (phenotyped) animals (File S2; please cite this report when using these data) as previously described (Aylor *et al.* 2011) using test arrays for the Mouse Diversity Array (Yang *et al.* 2009). Map distances were interpolated from the mm9/NCBI37 build of the mouse genome using the mousemapconverter tool (Cox *et al.* 2009; Shifman *et al.* 2006). Different markers assigned to the same base position were removed. Genotypes were further processed and checked for consistency using the prephappy perl script with a genotype error model of 0.01 (W. Valdar, personal communication).

### RNA preparation and oligonucleotide microarray processing

At 4 days after infection, mice were killed and lung tissue harvested and placed in RNAlater (Applied Biosystems/Ambion, Austin, TX) and stored at −80°. The tissues were subsequently homogenized, and RNA extracted as previously described (Pasieka *et al.* 2009). RNA samples were spectroscopically verified for purity, and the quality of the intact RNA was assessed using an Agilent 2100 Bioanalyzer. cRNA probes were generated from each sample by the use of an Agilent one-color Low Input Quick Amp Labeling Kit (Agilent Technologies, Santa Clara, CA). Individual cRNA samples were hybridized to Agilent mouse whole-genome oligonucleotide 4 × 44 microarrays according to manufacturer instructions. Samples from individual mice were evaluated to enable examination of animal-to-animal variation as part of the data analysis. Slides were scanned with an Agilent DNA microarray scanner, and the resulting images were analyzed using Agilent Feature Extractor version 8.1.1.1. The Agilent Feature Extractor software was used to perform image analysis, including significance of signal and spatial detrending and to apply a universal error model. For these hybridizations, the most conservative error model was applied. Raw data were then loaded into a custom-designed laboratory information management system (LIMS). Data were warehoused in a Labkey system (Labkey, Inc., Seattle, WA). Raw array data are available from GEO with accession GSE30506 and normalized intensity values are provided in File S3.

The Agilent arrays were background corrected by applying the Normal-Exponential convolution model (Irizarry *et al.* 2003) and normalized using quantile normalization (Bolstad *et al.* 2003) with the Agi4x44PreProcess Bioconductor package (Lopez-Romero 2011). The probes were filtered requiring that all probes meet specific QC requirements (probe intensity had to be found, well above background, not saturated, and not be nonuniformity or population outliers as defined by the standard parameters in Agi4x44PreProcess package) for all samples. Differential expression analysis was performed using the LIMMA Bioconductor package (Smyth 2005), and the false discovery rate was calculated using the qvalue Bioconductor package (Storey and Tibshirani 2003) (File S4). Probes were mapped to the mm9 genome using BLAT (Kent 2002) requiring at least 98% identity. Probes that did not map, mapped to multiple locations equally well, or contained a high confidence single nucleotide polymorphism (SNP) from one of the eight progenitor strains from the Sanger Institute/Wellcome Trust mouse sequencing project (Keane *et al.* 2011) in the probe sequence were excluded from analysis. There were 12,656 probes passing QC and not potentially impacted by a SNP (20,474 were uniquely mapped and passed QC, and, of these, 7818 were potentially impacted by SNPs). The Gene Ontology (GO) analysis was performed using the standard hypergeometric test from the GOstats Bioconductor package (Falcon and Gentleman 2007) with a universe consisting of the unique genes from the probes entered into the DE analysis. Only the Biological Process subset of the Gene Ontology was used for testing. The Benjamini and Yekutieli false discovery rate (FDR) (Benjamini and Yekutieli 2001) was computed for the *P*-value distribution for this analysis to address dependencies inherent from the hierarchical/nested structure of the GO categories.

### eQTL scan

Progenitor haplotype probabilities for each marker interval were inferred using the HAPPY R package (Mott *et al.* 2000) and an additive model. The number of generations was set to the average number of generations of the mice (seven generations). Significance of a given interval was assessed using a multiple partial F-test relating a simple single-locus additive model consisting of the expected haplotype contributions of each of the eight founders plus an intercept to an intercept-only model. A QR decomposition was performed on the design matrix of each marker interval for computational efficiency as has been described previously (Huang *et al.* 2009). Subsequently the probe-level residual sum of squares were computed. This avoided incurring the computational cost of decomposition plus residual computation for each phenotype as would typically be done if a linear model was fit separately for each phenotype and marker. Support intervals were computed using the 1.5 logarithm of odds (LOD) drop method that has been shown to be effective in dense marker sets (Dupuis and Siegmund 1999). LOD scores were computed for the marker intervals on the chromosome containing the most significant marker interval for the significant probes to reduce the computational burden. We denoted an eQTL as *trans*-acting if it was on a different chromosome compared with the probe. An eQTL was *cis*-acting if it resided on the same chromosome. The most significant logP value for each chromosome for each probe are provided in File S5.

### Allele effects

Allele effects were computed for the support intervals of each significant probe using the partial correlations of the HAPPY linear model fits (Aylor *et al.* 2011). To define the subset of strains contributing to the eQTL, we permuted the sample labels 1000 times and computed the allele effects for each. For each permutation run, we computed the Euclidean distance of the allele effects across the marker intervals and conducted average linkage hierarchical clustering recording the height of the top branch. The 95th percentile of the top branch heights was used as the cut point for the clustering of actual allele effects. Only those eQTL that formed at least two clusters were kept. The estimated allele effects are provided in File S6.

### SEM modeling

Adaptations of the five local SEM models previously described (Aten *et al.* 2008) were fit using the sem R package (Fox *et al.* 2010). To form a parsimonious SEM model, a forward variable-selection procedure was performed using the normalized intensity values for each probe and the eight expected haplotype contributions for the most significant marker interval of the probe. Our main model of interest was the “causal” model as illustrated in Figure S17A. This model was evaluated relative to the others using the *P*-value from the SEM model χ^2^ statistic divided by the maximum *P* value from the other four models and log10 transformed (essentially the LEO.NB.CPA score from the NEO R package) (Aten *et al.* 2008). The genes fitting the causal relationship best were chosen by requiring a score > 1, χ^2^ *P*-value > .05, root mean square error of approximation < 0.05, standardized root mean square residual < 0.1, comparative fit index > 0.9, and Wald *P*-value < 0.05. The GO analysis was performed as mentioned previously, using the probes remaining after application of the model comparison filters as input and the universe consisting of the significant probes for the LRI or HRI set. The expected haplotypic contributions for the eQTL analyzed using this method are provided in File S7.

### Real-time quantitative reverse transcription (qRT-PCR)

qRT-PCR was performed on lung homogenate samples from female mice from the eight founder strains killed at day 4 (D4) after PR8 infection. Three mice were assayed for each strain with each measurement performed in triplicate. The QuantiTect reverse transcription kit (QIAGEN Inc., Valencia, CA) was used to generate cDNA. qRT-PCR was run on an ABI 7500 PCR system, using TaqMan chemistry (Applied Biosystems, Foster City, CA). Gene expression assays specific to mouse cellular loci were purchased from Applied Biosystems. Twenty-one cellular loci were evaluated for expression patterns consistent with the allele effects. Five genes had custom probes designed for them (File S8). We subtracted the Ct value of each sample from the average value of the endogenous control (*Mfap1a*) and transformed each value X to X_*_log10(2) (File S9). For each gene, we fit a linear mixed effects model with strain as a fixed effect and incorporating a random intercept grouped by the strain samples. Using the allele effect clusters, we examined a contrast for each gene evaluating whether the mean values for each cluster were equivalent. Significance of these contrasts was determined by comparing the (χ^2^) *P*-value to the level after Bonferroni adjusting for the 17 comparisons (α = 0.05). We further required that the within-cluster ranks for the smallest clusters (or both if the same size) be consistent for both the allele effects and qPCR data for “complete” confirmation, otherwise we denoted the gene as a “partial” confirmation.

### Data analysis and visualization

All analysis was performed using Bioconductor version 2.6 (Gentleman *et al.* 2004), R version 2.11 (R Development Core Team 2011). Figures were generated using the ggplot2 R package (Wickham 2009). Mixed effects modeling was performed using lme4 (Bates *et al.* 2011) and linearHypothesis in car (Fox and Weisberg 2011) was used for evaluation of the contrasts.

## Results

To select for those genes likely involved in differential response to infection, we divided lung samples into two groups that represented mice from the extreme tails of the phenotypic distribution based upon weight change at 4 DPI and an IHC score based on the extent of infected cells throughout the lung (see the *Materials and Methods*; Figure 1). These animals were denoted as LRI (*n* = 18) or HRI (*n* = 26). The 44 arrays derived from these animals were preprocessed and normalized as described in the *Materials and Methods* and differential expression was assessed between the two groups. Of the analyzed probes, 1173 had normalized intensity values that were significantly up-regulated in HRI relative to LRI, and, similarly, 1498 were significantly up-regulated in LRI relative to HRI (q-value < 0.05 and a fold change ≥ 1.5 for both). An examination of biological process GO term enrichment in both categories showed that the genes up-regulated in LRI were overrepresented for processes that would be associated with normal growth and development such as “cell adhesion” (FDR = 0.008; Table S1). In contrast, the genes up-regulated in HRI were enriched for functional annotations related to the immune response such as “immune system process” (FDR = 3.97E^−21^; Table S1).

We performed eQTL scans for the probes in the HRI and LRI sets and required a stringent significance threshold based on a Bonferroni correction (α = 0.05) within each set as we considered each set to be its own analysis/eQTL scan. We found eQTL for 10 genes up-regulated in the HRI group that met the significance criteria (−log10 *P*-value > 9.631) and eQTL for 11 genes up-regulated in the LRI group (−log10 *P*-value > 9.738). The support intervals were 3 Mb on average and ranged from 409 Kb to 9.250 Mb. In total, across both comparisons, 20 probes were *cis*-regulated, and one was trans regulated (Table 1).

**Table 1:** Summary of the significant eQTL

Gene	Chr	Start, Mb	End, Mb	Size, Mb	logP	Type	Low Strains	High Strains	Status
A. High response to infection									
Gsdma	chr11	98.467	99.528	1.061	20.768	*cis*	NA	NA	N
LOC675467	chr14	20.239	20.648	0.409	12.766	*cis*	ABD	GC	S
							EFH		
Ifi27l2a	chr12	107.030	107.720	0.690	12.655	*cis*	G	ABCD	S
								EFH	
ENSMUSG00000052976	chr19	28.681	30.125	1.444	12.544	*cis*	FH	ABC	P
								DEG	
Dst	chr1	33.326	35.180	1.854	11.943	*cis*	ADEFH	BCG	F
Sik1	chr17	34.946	44.196	9.250	11.529	*cis*	BCEF	ADGH	P
AK144717	chr19	3.197	3.819	0.622	10.535	*cis*	NA	NA	N
NAP070792-1	chr7	114.496	118.040	3.544	10.436	*trans*	NA	NA	N
Senp5	chr16	29.637	31.571	1.934	10.19	*cis*	AFG	BCD	F
								EH	
Kcmf1	chr6	68.509	69.809	1.300	9.771	*cis*	F	ABCDEGH	S
B. Low response to infection									
Bmpr2	chr1	59.495	62.772	3.277	15.071	*cis*	ABC	FG	F
							DEH		
Tcf7l1	chr6	70.215	76.137	5.922	14.416	*cis*	FDG	ABC	F
								EH	
AK078430	chr3	131.067	133.998	2.931	13.914	*cis*	FG	ABC	P
								DEH	
Thnsl2	chr6	67.959	70.668	2.709	12.832	*cis*	GD	ABC	S
								EFH	
AK153595	chr17	5.220	7.352	2.132	12.013	*cis*	GD	ABC	S
								EFH	
D930030005Rik	chr7	73.676	77.803	4.127	11.056	*cis*	ABC	FG	P
							DEH		
BC022687	chr12	112.813	114.454	1.641	11.017	*cis*	BDC	AEF	S
								GH	
Sh3gl3	chr7	87.808	91.022	3.214	10.721	*cis*	ABC	FG	S
							DEH		
Clec16a	chr16	6.328	10.873	4.545	10.446	*cis*	B	ACDE	S
								FGH	
Pde7a	chr3	26.126	27.769	1.643	10.358	*cis*	ABC	F	F
							DEGH		
1190007I07Rik	chr10	76.093	84.837	8.744	10.011	cis	NA	NA	N

An eQTL support interval (NCBI37/mm9) is defined by a 1.5 LOD drop from the maximum LOD score at the marker with the maximum –log10 *P*-value (logP column). Type indicates whether the eQTL was on the same chromosome as the gene (*cis*) or not (*trans*). The low strain column indicates those strains that had lower allele effects relative to the strains in the high strain column. Strains were one of A (A/J), B (C57BL/6J), C (129S1/SvImJ), D (NOD/ShiLtJ), E (NZO/HILtJ), F (CAST/EiJ), G (PWK/PhJ) and H (WSB/EiJ) or NA indicating not applicable. The status column indicates whether the allele effects for that eQTL were consistent (S) with the allele's effects in inbred founder strains, partially consistent (P), not tested (N), or inconsistent (F). eQTL, expression quantitative trait loci.

**Figure 2:**
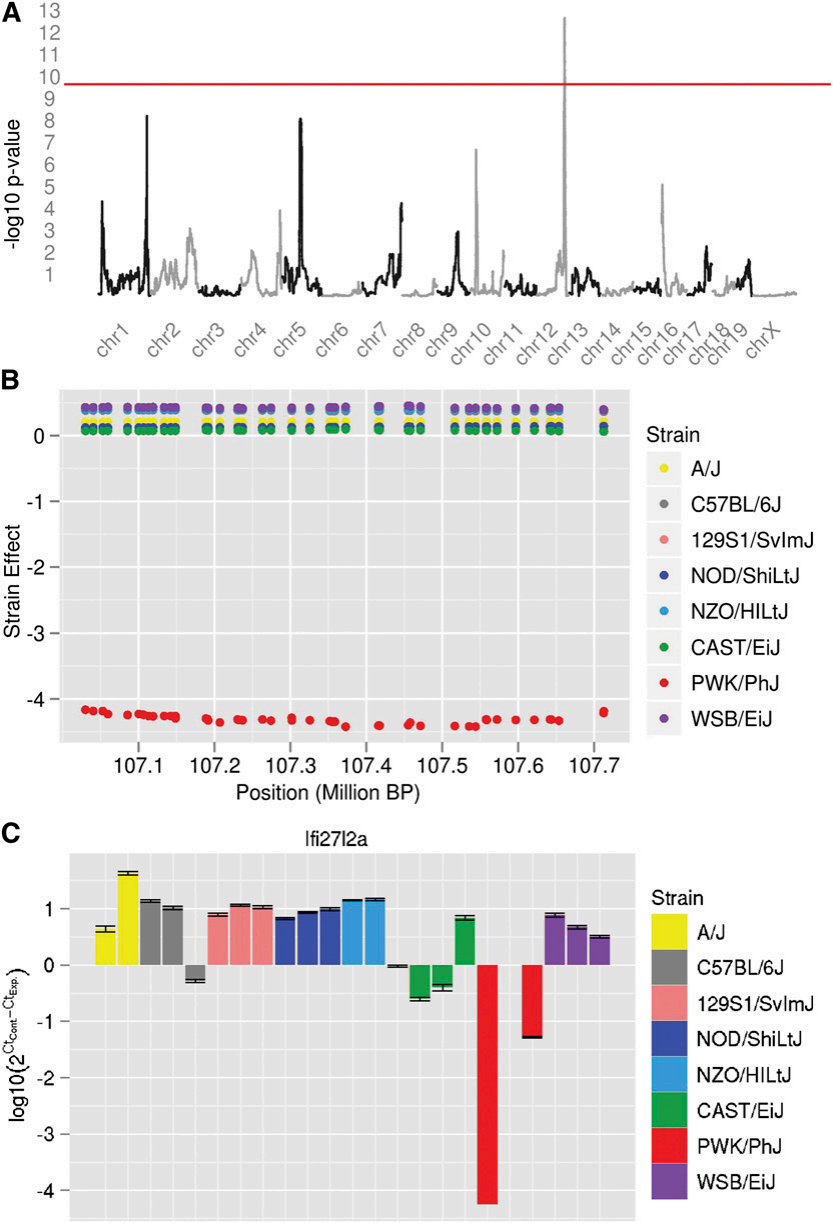
Ifi27l2a is a significantly *cis*-regulated gene with expression differences driven mainly by the PWK/PhJ allele. (A) A significant eQTL is located in distal Chr 12 (LOD = 18.7; 170.030−170.720 Mb). (B) The allele effects for the markers in the support interval indicate pre-CC mice with an allele inherited from the PWK/PhJ founder strain expressed *Ifi27I2a* at a lower level than the rest of the population. (C) The same pattern of allele effects was confirmed by qPCR in three animals from each of the eight founder strains, as we expect from a *cis*-regulated eQTL.

We performed confirmation experiments in a set of mice from the eight inbred founder strains. Mice were infected with influenza A virus in the same manner as the pre-CC mice and we assayed expression of candidate gene via qPCR at 4 DPI (see *Materials and Methods*). Of the 21 genes, assays failed for at least one progenitor strain for *Gsdma* and *NAP0707921-1*. In addition, the allele effects for two additional genes, *AK144717* and *1190007107Rik*, could not be separated into distinct clusters on the basis of our clustering strategy. We successfully examined 17 genes and found expression values completely consistent with the eQTL allele effects (see the *Materials and Methods*) for eight (Figure S1, Figure S2, Figure S3, Figure S4, Figure S5, Figure S6, and Figure S7; Figure 2). whereas an additional four were partially consistent (Figure S8, Figure S9, Figure S10, and Figure S11). *Ifi27l2a*, is a gene previously implicated in the immune response (Labrada *et al.* 2002), which was observed to be up-regulated in the HRI group and is expressed much lower in the PWK/PhJ strain than in the other strains, consistent with the observed allele effects from the pre-CC (Figure 2). Three poorly characterized genes (*LOC675467*, *AK153595*, and *BC022687*) were also confirmed as having expression patterns completely consistent with allele effects, indicating that they may be potentially important in the resistance or susceptibility to Influenza A infection. The five genes that did not conform to expected allele effects are shown in Figure S12, Figure S13, Figure S15, Figure S15, and Figure S16.

We performed a local SEM analysis to infer potential causal relationships between each of the eight completely confirmed genes and the other remaining genes within their respective response groups. We first selected the expected haplotype contributions to use in the SEM model through forward variable selection (Table S2) and then generated and compared the five possible models summarizing our proposed variable relationships (*Materials and Methods* and Figure S17). Of the eight genes, seven were found to have at least one putative causative relationship resulting in a total of 166 relationships (Table S3). Because *Ifi27l2a*, *Sh3gl3*, and *Kcmf1* had relatively large number of reactive (“downstream”) genes (25, 108, and 24, respectively), we examined functional enrichment of the potential “downstream” genes for these genes. We found that for *Ifi27l2a*, the most enriched categories related to signal transduction and transportation (Table S4A), whereas for *Sh3gl3* the top categories related to cellular growth and development (Table S4B) and for *Kcmf1* they involved metabolic processes (Table S4C). In contrast, for *Thnsl2*, *AK153595*, *BC022687*, and *Clec16a*, there were too few reactive genes for testing for GO enrichment, and no direct associations were noted in either the literature or through functional annotation, indicating that this SEM approach may provide a way to identify and tease apart currently uncharacterized regulatory mechanisms.

## Discussion

Transcriptional profiles related to extreme differences in host responses to infectious diseases can likely provide insights into host genetic contributions to susceptibility and protection. In this study, we used a genetically diverse panel of mice, the pre-CC population infected with influenza A virus, to identify mice that showed extreme (high and low) responses to infection based on two clinical readouts. We then identified transcripts differentially expressed between the high and low response groups. By focusing on differentially expressed genes, we successfully identified and validated eQTL controlling several of these transcripts, which were then used for downstream modeling in order to begin to infer the underlying casual relationships in the host response regulatory network.

At 4 DPI with influenza virus, approximately 21% of all genes were differentially expressed between the high and low responders to infection. This finding is impressive, given both the conservative criteria we used for statistical significance and the smaller sample size due to the strict phenotypic requirements. However, the focus on the extreme tails of the phenotype distribution provided additional power for detecting transcripts implicated in differential host response. These putative candidates belonged to a variety of functional categories, including immune responses, tissue regeneration, and cellular adhesion. For those genes up-regulated in the HRI samples, membership of genes related to immune function is unsurprising, given that these individuals are experiencing uncontrolled viral infection, as well as severe disease. Similarly, the increases in cellular, tissue, vascular, and other growth responses in the HRI class are reasonable in light of tissue repair following apoptotic and/or necrotic cell death during infection. In contrast, those genes up-regulated in the LRI were primarily enriched for cellular adhesion. Cell adhesion pathways have been implicated (Herold *et al.* 2006) in mediating the host inflammatory cell infiltration response to Influenza A virus, and it was suggested they may be key for modulating the severe histopathology observed in acute influenza virus pneumonia.

To better understand how host genetic factors contribute to the differential transcriptional profiles of the HRI and LRI classes, we used very stringent criteria to identify 21 high-confidence eQTL, host genome regions directly controlling expression levels of differentially expressed genes between HRI and LRI mice. The preponderance of *cis*-regulated loci identified is not surprising, given the limited power to detect trans eQTL in these mapping studies (Aylor *et al.* 2011). Those genes with significant host genetic control that are differentially regulated between HRI and LRI are predominantly related to immunity and/or the response to infection. Although none has been identified as important in influenza infection, it is not surprising that we identified and confirmed genes relating to immune function in our eQTL scans. *Ifi27l2a* (also referred to as *Isg12*) is nuclear localized (Martensen *et al.* 2001) and regulates the function of the orphan nuclear receptor NR4A1 protein, especially in response to vascular inflammation (Papac-Milicevic 2007), a likely byproduct of influenza infection. Similarly, *Clec16a* is a member of the C-type lectin family of genes, which are considered critical for tailoring immune responses to pathogens, as they can trigger distinct signaling pathways after pathogen binding (Geijtenbeek and Gringhuis 2009). These results suggest that specific modulations of the extent and nature of the immune response, as well as the development of an adaptive immune response might all contribute to the differential disease phenotypes observed.

It is less clear how some of the other validated eQTL contribute to influenza virus response, particularly those unannotated/poorly annotated transcripts where we cannot postulate mechanisms. For example, *Sh3gl3* (or endophilin A3) has been observed binding with a number of proteins, including *Mta1*, and may be involved in endocytosis (Aramaki *et al.* 2005), although the role this might play in infection is not clear. Similarly, the role of *Thnsl2* as a dephosphatase on phosphorylated amino acids (Donini *et al.* 2006) does not immediately suggest a role in viral infection, Nevertheless, it is clear that further work must be done to clarify the potential roles these genes have in mediating any potential host responses to infection, although our conservative approach strongly suggests that they do in fact play roles in the host response

The relative roles of components of the immune system in promoting protective and pathologic responses to influenza A virus infection has long been debated, with a clear result that uncontrolled immune responses can contribute to pathology (Kash *et al.* 2006). We would clearly expect increased transcriptional levels of immune gene transcription in the HRI class. However, when looking at the eQTL identified in our analysis, many of the genes influenced by eQTL in both the high and low response groups have relationships to immune functions. These results suggest that up or down regulation of these various transcripts might be of critical importance in contributing to protection and pathology in response to influenza infection.

In some ways, it is surprising that polymorphisms at the *Mx1* gene, which segregate in the pre-CC population, do not contribute to the identified eQTL. Especially because *Mx1* is a known potent anti-influenza gene (Staeheli and Haller 1987; Zimmermann *et al.* 2011). However, it is clear that *Mx1* does play a role in determining the classification of mice into the HRI and LRI classes, as 24/26 animals in the HRI group were homozygous for a variant of *Mx1* lacking activity, while none of the animals in the LRI class had a non-functional *Mx1* (*P*-value = 2.2 × 10^−16^; Fisher's exact test). Based upon our data, the effect *of Mx1* is minimal at this time post infection, with *cis*-factors being much more critical in determining the actual transcript levels of various differentially expressed genes. This is most clearly shown by the fact that the region containing the *Mx1* locus on chromosome 16 contributed no significant trans eQTL.

In this study, we were able to identify eQTL and confirm that a subset had expression patterns consistent with the expected allele effects in the eight inbred founder strains. We note that the validation in the inbred founder strains assumes that the effect of *cis* eQTL is not altered by genetic background and that violations of these assumptions could lead to a higher false negative rate in the validation. In the future, it will be possible given the reproducible nature of the Collaborative Cross recombinant inbred lines (The Complex Trait Consortium 2004; Collaborative Cross Consortium 2012) to perform a replication experiment using the lines that contain the regulatory alleles for the genes identified here.

One way to further understand the roles that eQTL play in an overall transcriptional environments is to utilize causal modeling. In this study, we used a SEM modeling approach to identify additional transcripts that are potentially influenced indirectly by a loci's effect on a given transcript, focusing on the eight transcripts that were validated in the parental lines. We were able to postulate “causal” relationships between seven of the eight confirmed genes and other genes that were present in their respective infection groups. GO enrichment for the inferred reactive partners of three of the genes with the greatest number of relationships indicated the presence of relatively diverse functionality. Interestingly, some evidence exists that *Clec16a* and its two putative partners may be members of the same pathway (Szklarczyk *et al.* 2011), suggesting that this approach does allow us to identify interacting partners.

Previous investigators have utilized SEM modeling, but have considered simpler mouse crosses (Aten *et al.* 2008). Here, we expand these approaches to work with eQTL mapping in genetically complex populations, such as the Collaborative Cross or heterogenous stock mice. Instead of utilizing only the additively encoded genotypes, our local SEM models work directly work with the expected haplotype contributions from each of founder strains. Such contributions can be derived from any method such as HAPPY (Mott *et al.* 2000) or GAIN (Liu *et al.* 2010). Our approach allows for further identification of potential regulatory relationships between genes, as well as further suggestions of the subnetworks that might play important roles in promoting either a protective/low response or pathologic/high response to influenza infection. We further note that although we only focused on a single marker analysis in this work (largely because of sample size concerns), additional strategies could be carried out utilizing multiple genetic markers, further enhancing our ability to disentangle important sub-networks.

The genetic variation present in the Collaborative Cross lines allows for not only the mapping of genetic loci contributing to differential phenotypes (in this case, expression), but also for the modeling of a genetically diverse population. Indeed, as our data suggest, the extreme genetic diversity present in the Collaborative Cross makes it likely that genes poorly annotated based on the classic inbred strains (such as C57BL/6J) might receive increased attention with regard to their importance in complex traits. More importantly, because the Collaborative Cross lines recapitulate aspects of a genetically diverse population, we will begin to have the ability to identify underlying causal variants, and reach the full potential of systems genetics approaches

The use of a very focused systems genetics framework allowed us to identify high-confidence candidates implicated in differential host response, even with limited sampling. From this work, we are able to begin to elucidate the causal relationships in the underlying regulatory networks that will guide future perturbation studies in order to identify targets that modulate host response in Influenza and could assist future therapeutic development.

## Supplementary Material

Supporting Information
