# Supplementary material for: Expression Quantitative Trait Loci for Extreme Host Response to Influenza A in Pre-Collaborative Cross Mice
Source: G3 (Bethesda). 2012 Feb 1;2(2):213–21. doi: 10.1534/g3.111.001800 (PMC3284329; doi:10.1534/g3.111.001800)
Supplement: Supporting Information [file supp_2_2_213__index.html]

Supporting Information 

# Expression Quantitative Trait Loci for Extreme Host Response to Influenza A in Pre-Collaborative Cross Mice

## Supporting Information for Bottomly *et al.*, 2012

**Files in this Data Supplement:**

- Supporting Information - Figures S1-S17 and Tables S1-S4 (PDF, 11.1 MB)
- Figure S1 - Allele effect and qPCR plots. Allele effect and qPCR plots for the 7 remaining genes (Ifi27l2a is shown in Figure 2) with allele effects completely consistent with the qPCR data are shown in Figures S1-S7. Similarly the partially consistent genes are shown in Figures S8-S11 and those that failed to confirm are shown in Figures S12-16 (PDF, 695 KB)
- Figure S2 - (PDF, 703 KB)
- Figure S3 - (PDF, 605 KB)
- Figure S4 - (PDF, 520 KB)
- Figure S5 - (PDF, 655 KB)
- Figure S6 - (PDF, 657 KB)
- Figure S7 - (PDF, 568 KB)
- Figure S8 - (PDF, 653 KB)
- Figure S9 - (PDF, 625 KB)
- Figure S10 - (PDF, 834 KB)
- Figure S11 - (PDF, 666 KB)
- Figure S12 - (PDF, 701 KB)
- Figure S13 - (PDF, 790 KB)
- Figure S14 - (PDF, 669 KB)
- Figure S15 - (PDF, 773 KB)
- Figure S16 - (PDF, 654 KB)
- Figure S17 - An example of the path model diagrams for the 5 specified structural equation models (PDF, 89 KB)
- Table S2 - Strain contributions chosen by forward variable selection (PDF, 45 KB)
- Table S4 - GO Categories for the genes reactive to Ifi27l2a , Sh3gl3 and Kcmf1 (PDF, 71 KB)
- Table S1 - Enriched GO terms for up-regulated genes in each extreme phenotypic group (.csv, 4 KB)
- Table S3 - Local SEM inferred causal relationships (.csv, 7 KB)
- File S1 - supporting data (.csv, 2 KB)
- File S2 - supporting data (.zip, 6. 5 MB)
- File S3 - supporting data (.zip, 16.3 KB)
- File S4 - supporting data (.csv, 898 KB)
- File S5 - supporting data (.csv, 1 MB)
- File S6 - supporting data (.csv, 760 KB)
- File S7 - supporting data (.csv, 70 KB)
- File S9 - supporting data (.csv, 85 KB)
