# Supplementary material for: Expression Quantitative Trait Loci for Extreme Host Response to Influenza A in Pre-Collaborative Cross Mice
Source: G3 (Bethesda). 2012 Feb 1;2(2):213–21. doi: 10.1534/g3.111.001800 (PMC3284329; doi:10.1534/g3.111.001800)
Supplement: Supporting Information [file supp_2.2.213_001800SI.pdf]

A.

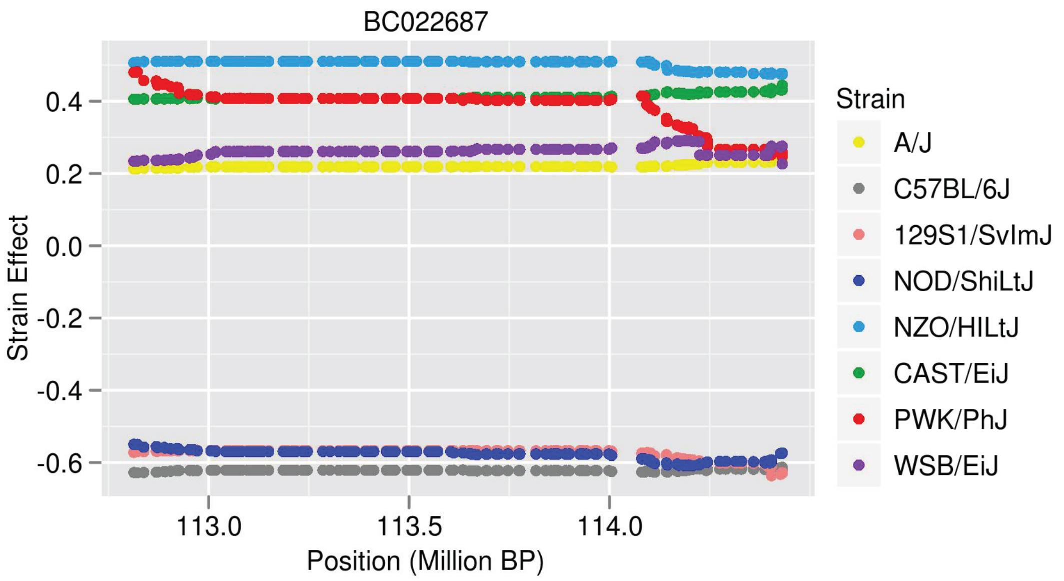
BC022687
Strain Effect
0.4
0.2
0.0
-0.2
-0.4
-0.6
113.0
113.5
114.0
Position (Million BP)
Strain
A/J
C57BL/6J
129S1/SvImJ
NOD/ShiLtJ
NZO/HILtJ
CAST/EiJ
PWK/PhJ
WSB/EiJ

B.

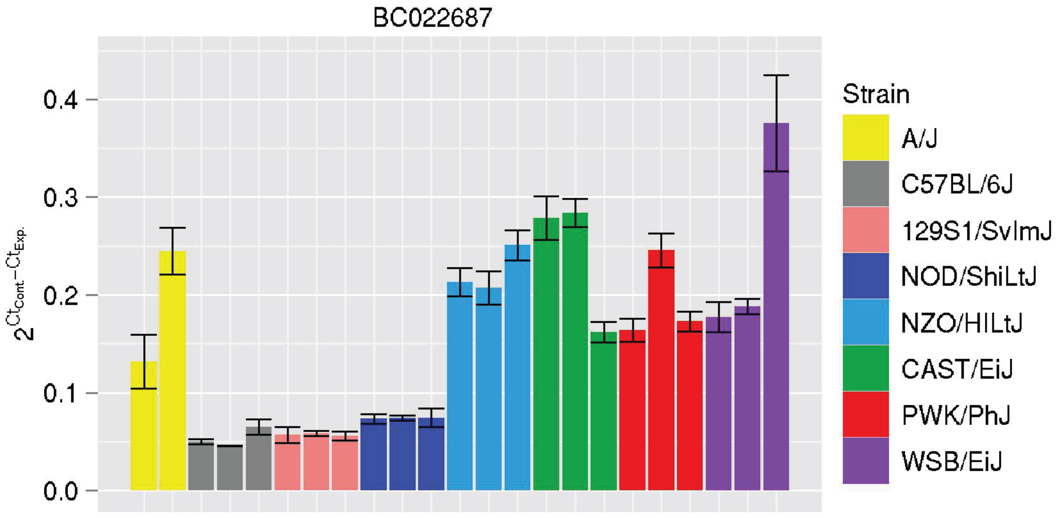
BC022687
$2^{Ct_{Cont.}-Ct_{Exp.}}$
0.4
0.3
0.2
0.1
0.0
Strain
A/J
C57BL/6J
129S1/SvImJ
NOD/ShiLtJ
NZO/HILtJ
CAST/EiJ
PWK/PhJ
WSB/EiJ

Figure S1

A.

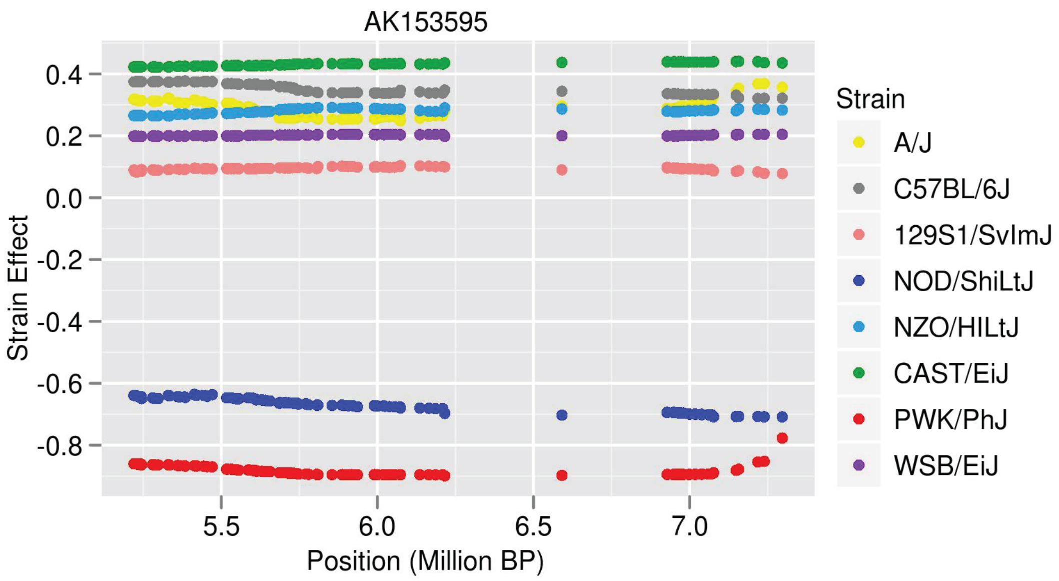
AK153595
Strain Effect
0.4
0.2
0.0
-0.2
-0.4
-0.6
-0.8
5.5
6.0
6.5
7.0
Position (Million BP)
Strain
A/J
C57BL/6J
129S1/SvImJ
NOD/ShiLtJ
NZO/HILtJ
CAST/EiJ
PWK/PhJ
WSB/EiJ

B.

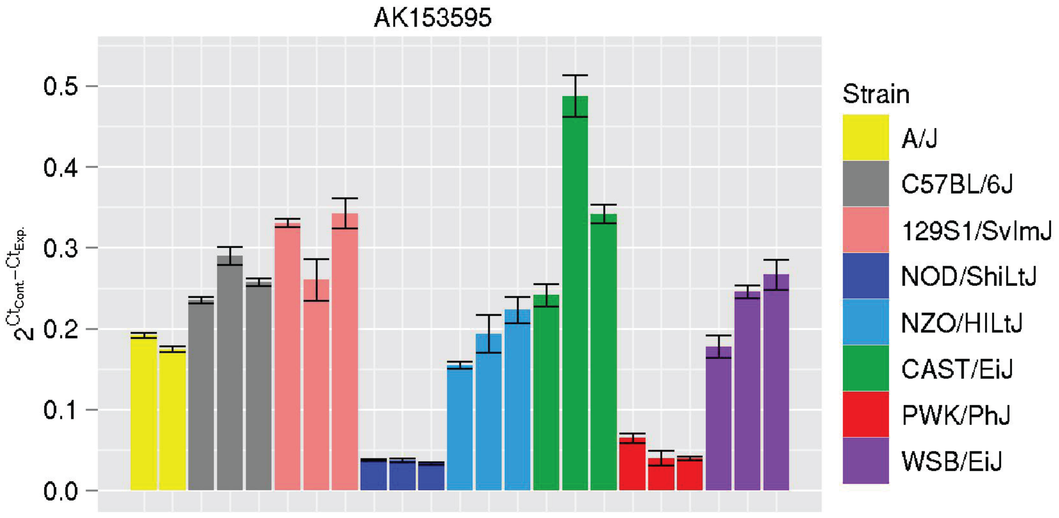
AK153595
$2^{Ct_{Cont.}-Ct_{Exp.}}$
0.5
0.4
0.3
0.2
0.1
0.0
Strain
A/J
C57BL/6J
129S1/SvImJ
NOD/ShiLtJ
NZO/HILtJ
CAST/EiJ
PWK/PhJ
WSB/EiJ

Figure S2

A.

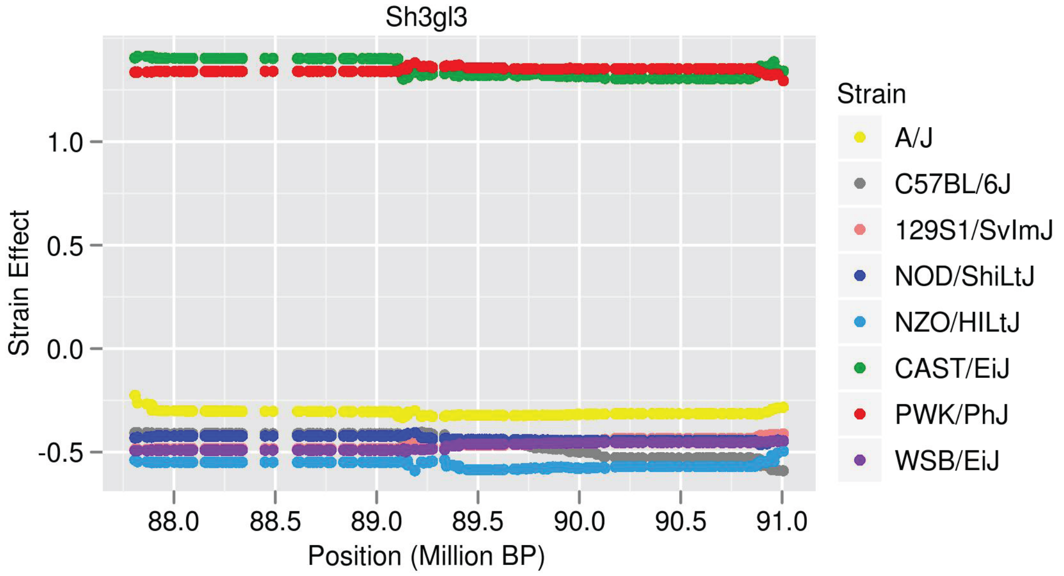
Sh3gl3
Strain Effect
1.0
0.5
0.0
-0.5
88.0
88.5
89.0
89.5
90.0
90.5
91.0
Position (Million BP)
Strain
A/J
C57BL/6J
129S1/SvImJ
NOD/ShiLtJ
NZO/HILtJ
CAST/EiJ
PWK/PhJ
WSB/EiJ

B.

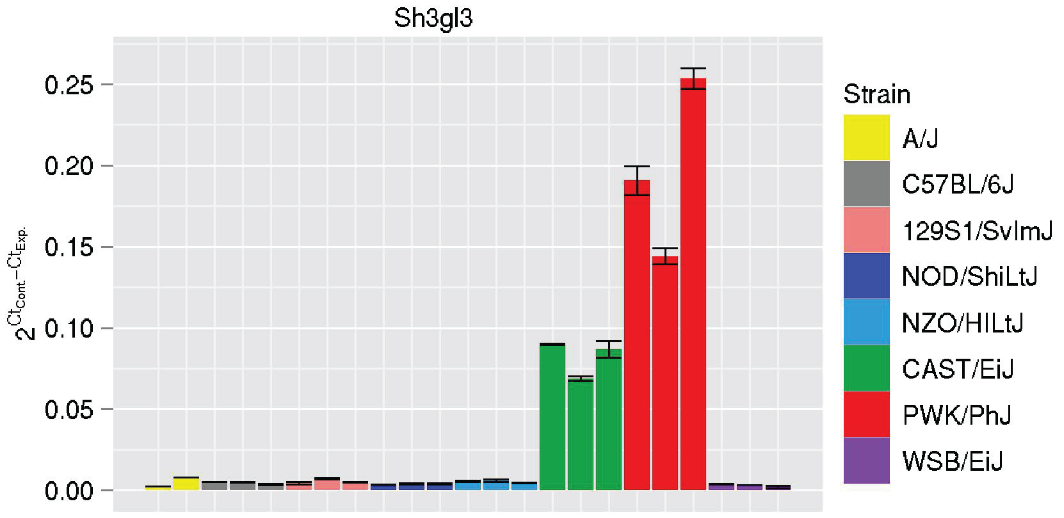
Sh3gl3
0.25
0.20
0.15
0.10
0.05
0.00
Strain
A/J
C57BL/6J
129S1/SvImJ
NOD/ShiLtJ
NZO/HILtJ
CAST/EiJ
PWK/PhJ
WSB/EiJ

Figure S3

A.

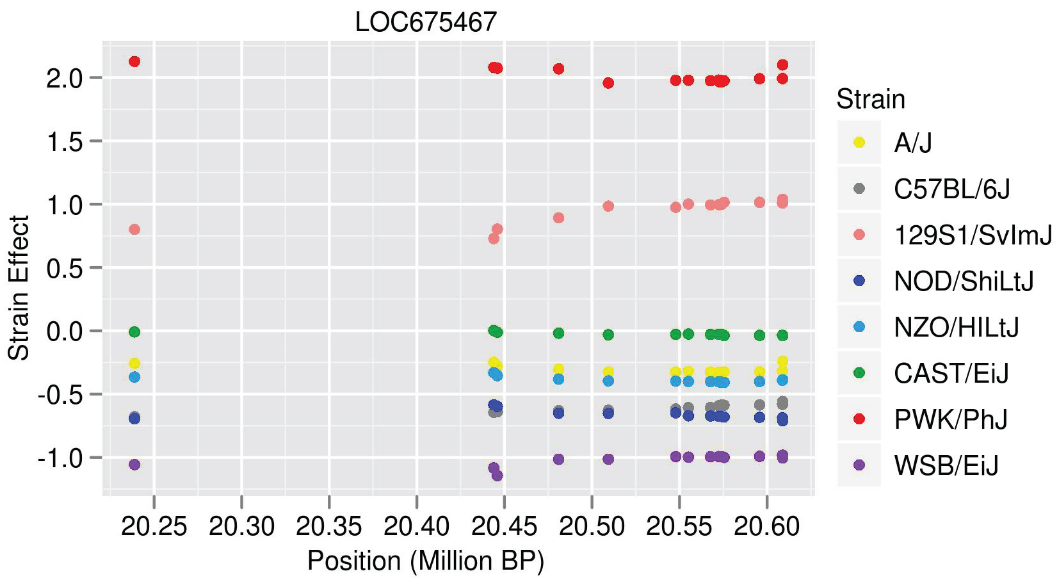
LOC675467
Strain Effect
2.0
1.5
1.0
0.5
0.0
-0.5
-1.0
20.25
20.30
20.35
20.40
20.45
20.50
20.55
20.60
Position (Million BP)
Strain
A/J
C57BL/6J
129S1/SvImJ
NOD/ShiLtJ
NZO/HILtJ
CAST/EiJ
PWK/PhJ
WSB/EiJ

B.

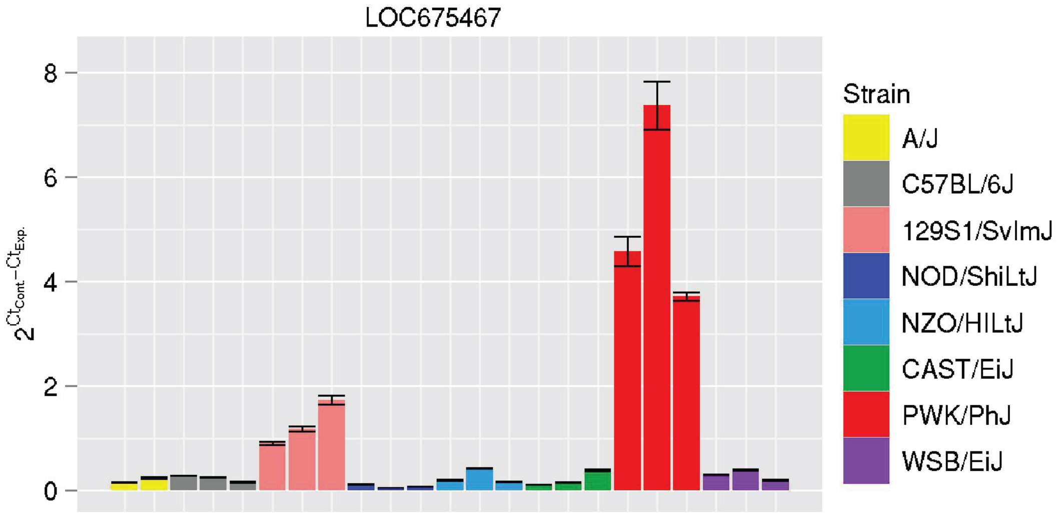
LOC675467
$2^{Ct_{Cont.}-Ct_{Exp.}}$
8
6
4
2
0
Strain
A/J
C57BL/6J
129S1/SvImJ
NOD/ShiLtJ
NZO/HILtJ
CAST/EiJ
PWK/PhJ
WSB/EiJ

Figure S4

A.

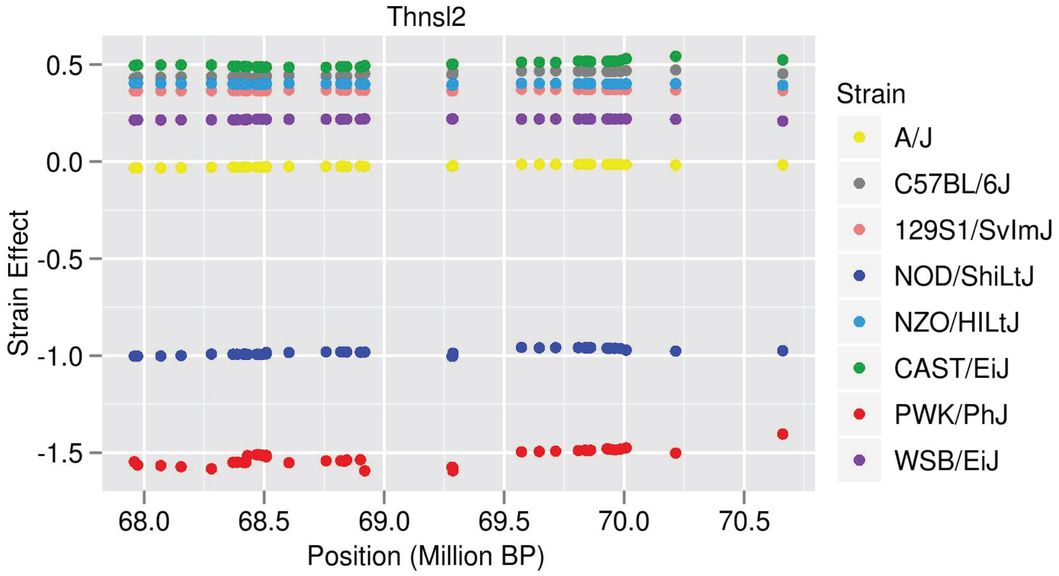
Thnsl2
0.5
0.0
-0.5
-1.0
-1.5
Strain Effect
68.0
68.5
69.0
69.5
70.0
70.5
Position (Million BP)
Strain
A/J
C57BL/6J
129S1/SvImJ
NOD/ShiLtJ
NZO/HILtJ
CAST/EiJ
PWK/PhJ
WSB/EiJ

B.

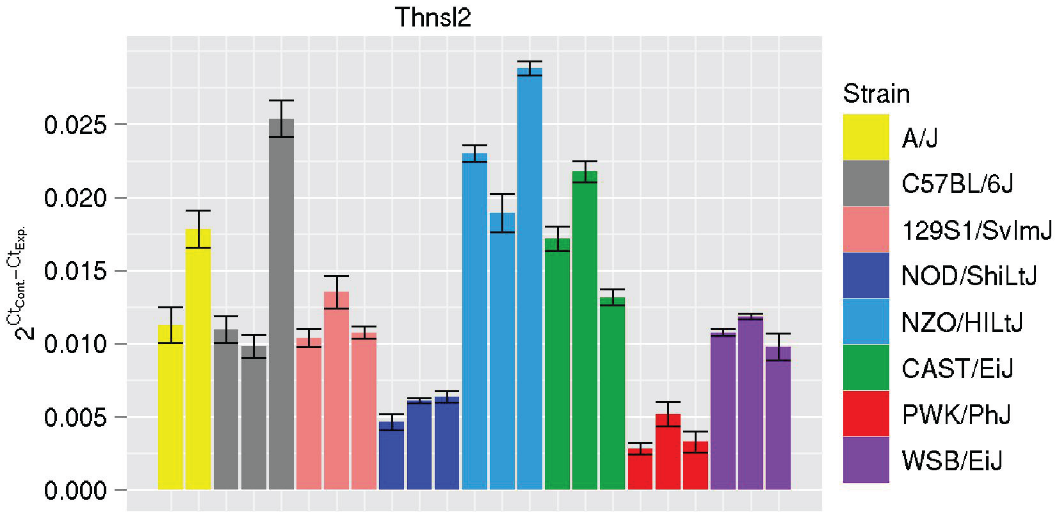
Thnsl2
0.025
0.020
0.015
0.010
0.005
0.000
$2^{Ct_{Cont.}-Ct_{Exp.}}$
Strain
A/J
C57BL/6J
129S1/SvImJ
NOD/ShiLtJ
NZO/HILtJ
CAST/EiJ
PWK/PhJ
WSB/EiJ

Figure S5

A.

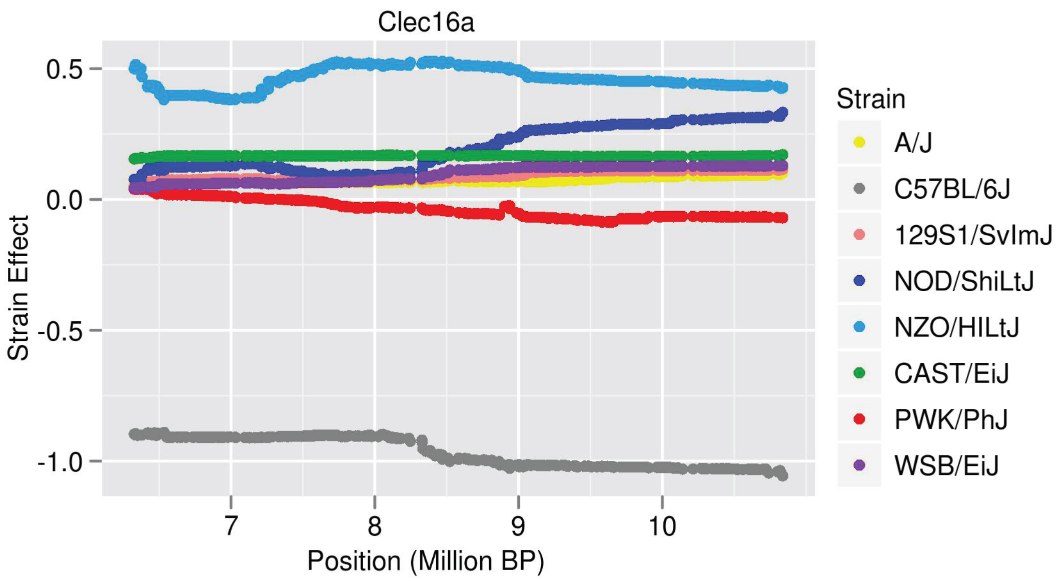
Clec16a
Strain Effect
0.5
0.0
-0.5
-1.0
7
8
9
10
Position (Million BP)
Strain
A/J
C57BL/6J
129S1/SvImJ
NOD/ShiLtJ
NZO/HILtJ
CAST/EiJ
PWK/PhJ
WSB/EiJ

B.

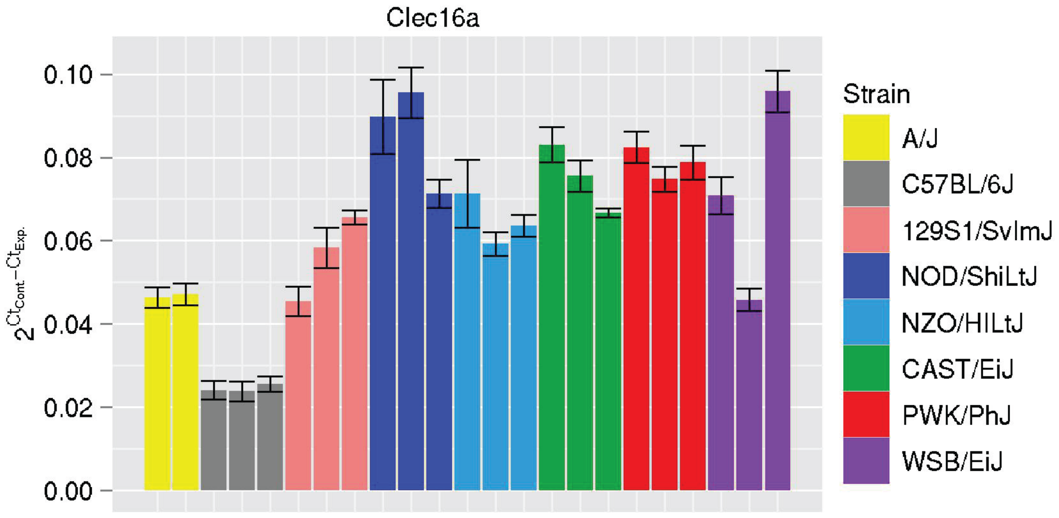
Clec16a
$2^{Ct_{Cont.}-Ct_{Exp.}}$
0.10
0.08
0.06
0.04
0.02
0.00
Strain
A/J
C57BL/6J
129S1/SvImJ
NOD/ShiLtJ
NZO/HILtJ
CAST/EiJ
PWK/PhJ
WSB/EiJ

Figure S6

A.

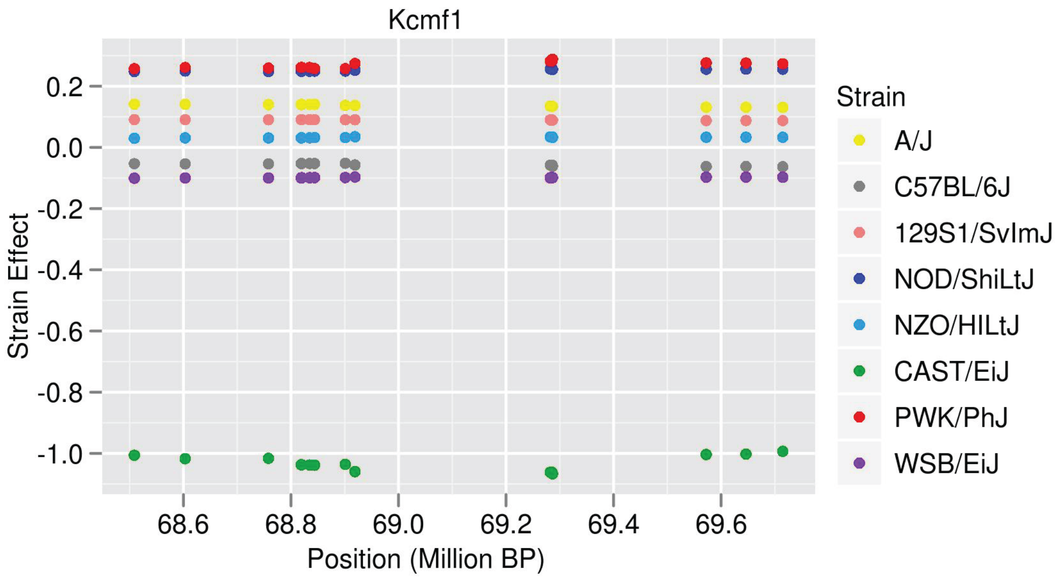
Kcmf1
Strain Effect
0.2
0.0
-0.2
-0.4
-0.6
-0.8
-1.0
68.6
68.8
69.0
69.2
69.4
69.6
Position (Million BP)
Strain
A/J
C57BL/6J
129S1/SvImJ
NOD/ShiLtJ
NZO/HILtJ
CAST/EiJ
PWK/PhJ
WSB/EiJ

B.

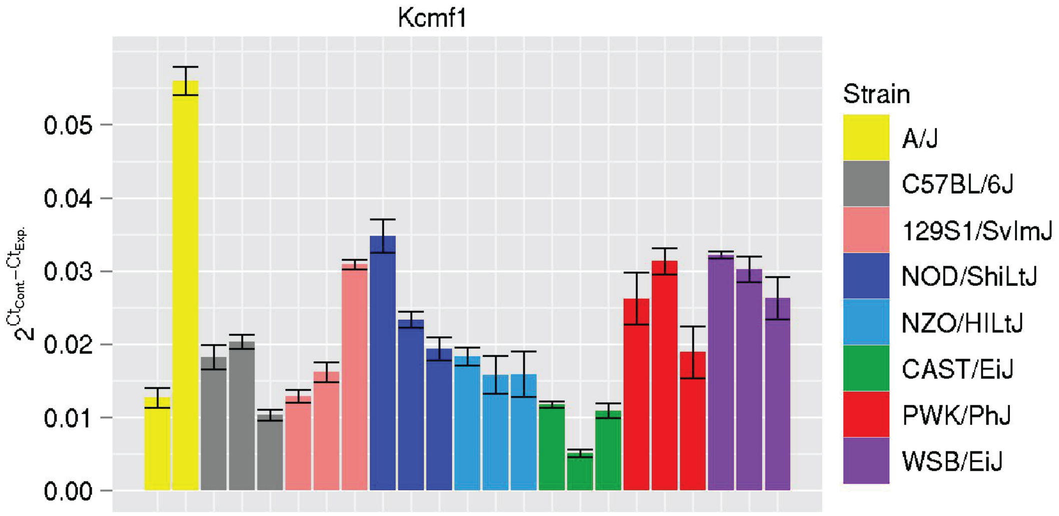
Kcmf1
$2^{Ct_{Cont.}-Ct_{Exp.}}$
0.05
0.04
0.03
0.02
0.01
0.00
Strain
A/J
C57BL/6J
129S1/SvImJ
NOD/ShiLtJ
NZO/HILtJ
CAST/EiJ
PWK/PhJ
WSB/EiJ

Figure S7

A.

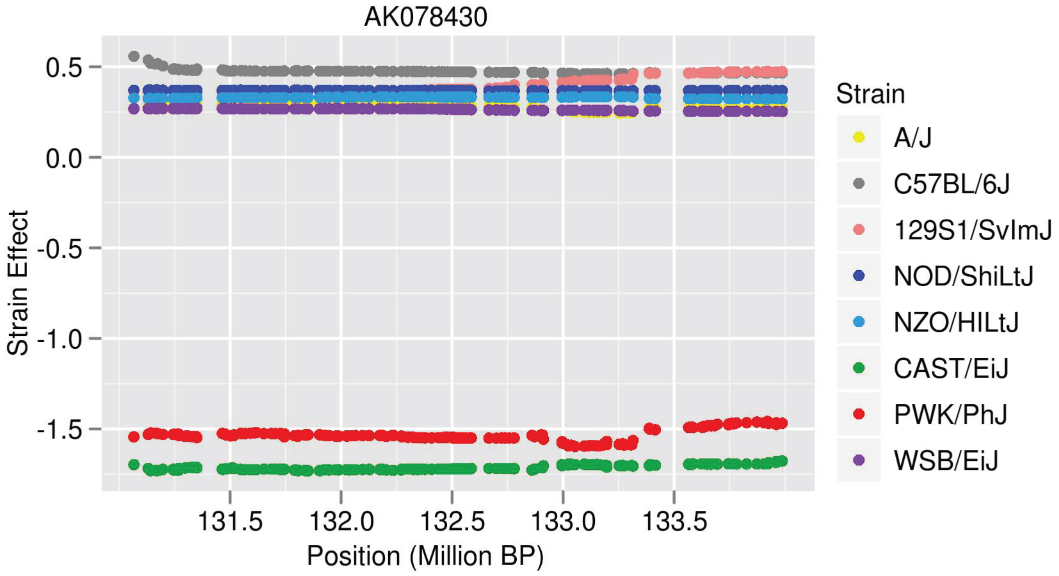
AK078430
0.5
0.0
-0.5
-1.0
-1.5
Strain Effect
131.5
132.0
132.5
133.0
133.5
Position (Million BP)
Strain
A/J
C57BL/6J
129S1/SvImJ
NOD/ShiLtJ
NZO/HILtJ
CAST/EiJ
PWK/PhJ
WSB/EiJ

B.

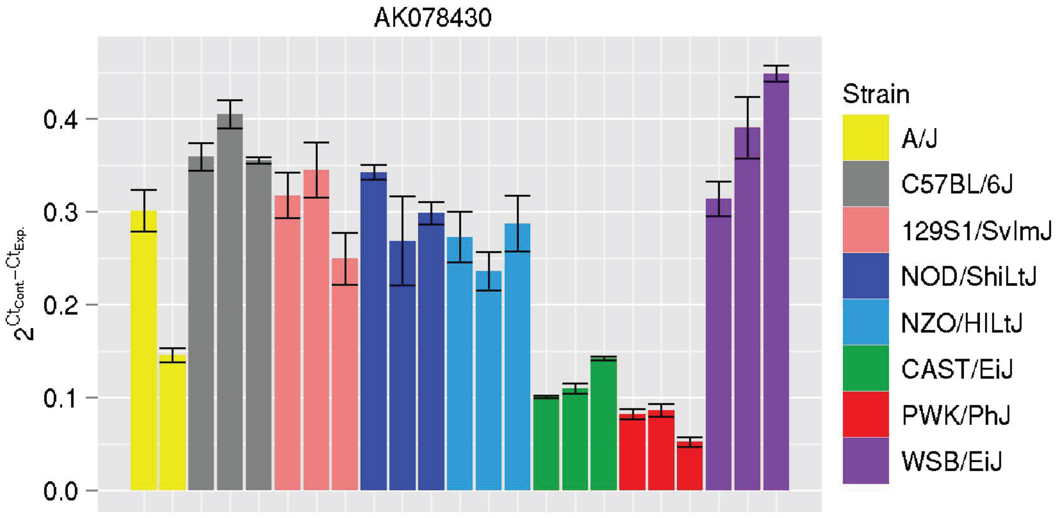
AK078430
0.4
0.3
0.2
0.1
0.0
$2^{Ct_{Cont.}-Ct_{Exp.}}$
Strain
A/J
C57BL/6J
129S1/SvImJ
NOD/ShiLtJ
NZO/HILtJ
CAST/EiJ
PWK/PhJ
WSB/EiJ

Figure S8

A.

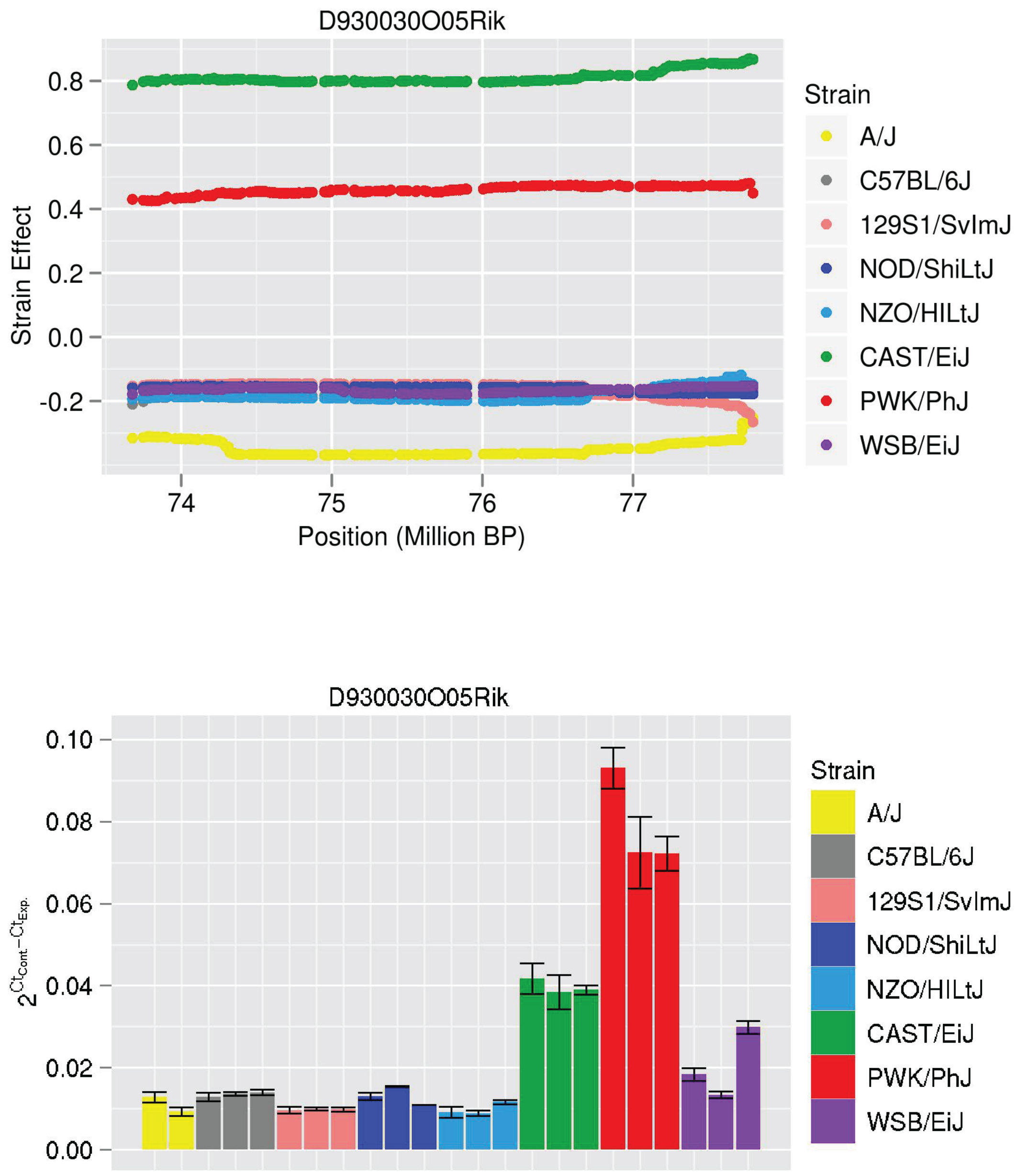
D930030O05Rik
Strain Effect
0.8
0.6
0.4
0.2
0.0
-0.2
74
75
76
77
Position (Million BP)
Strain
A/J
C57BL/6J
129S1/SvImJ
NOD/ShiLtJ
NZO/HILtJ
CAST/EiJ
PWK/PhJ
WSB/EiJ
B.
D930030O05Rik
0.10
0.08
0.06
0.04
0.02
0.00
Strain
A/J
C57BL/6J
129S1/SvImJ
NOD/ShiLtJ
NZO/HILtJ
CAST/EiJ
PWK/PhJ
WSB/EiJ

Figure S9

A.

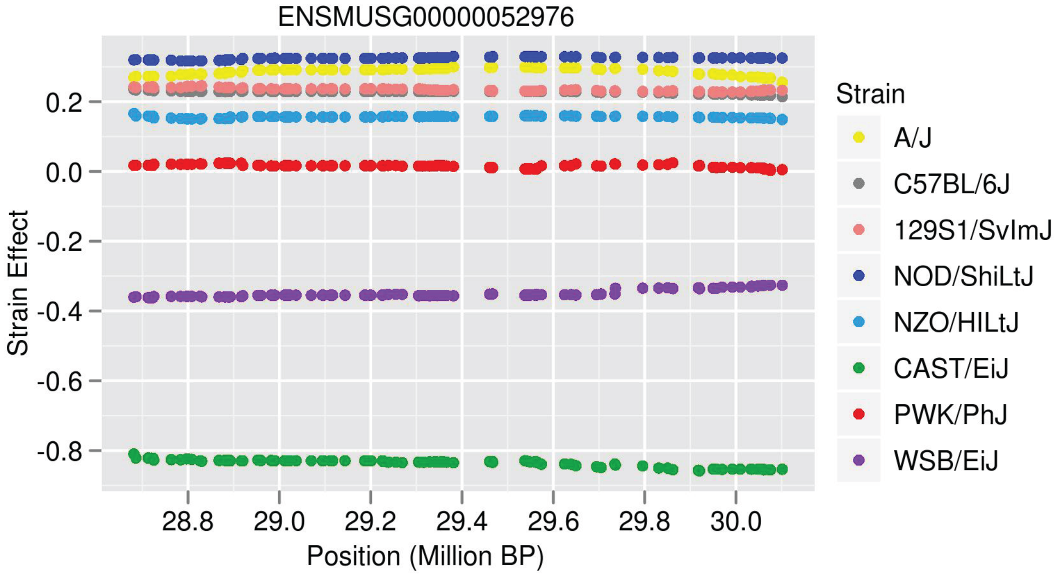

B.

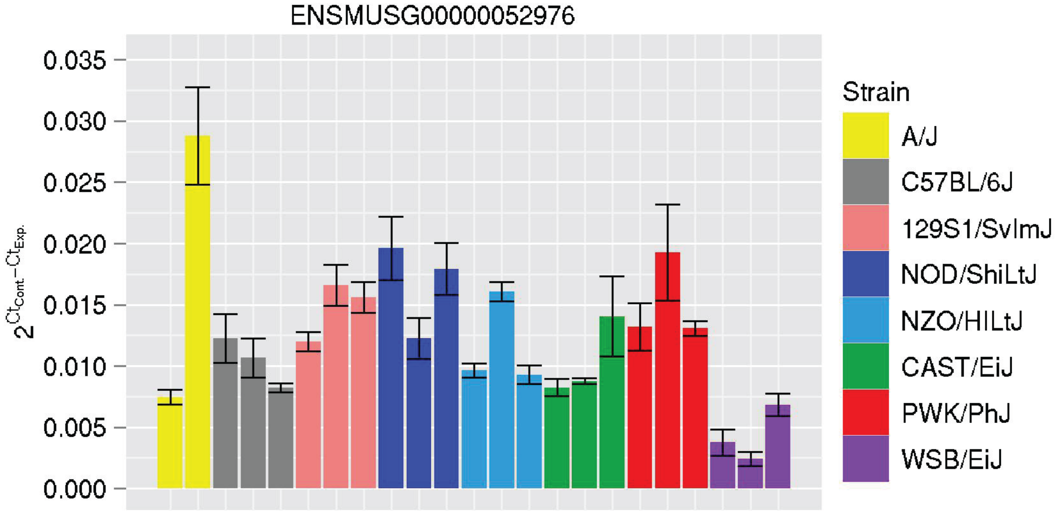

Figure S10

A.

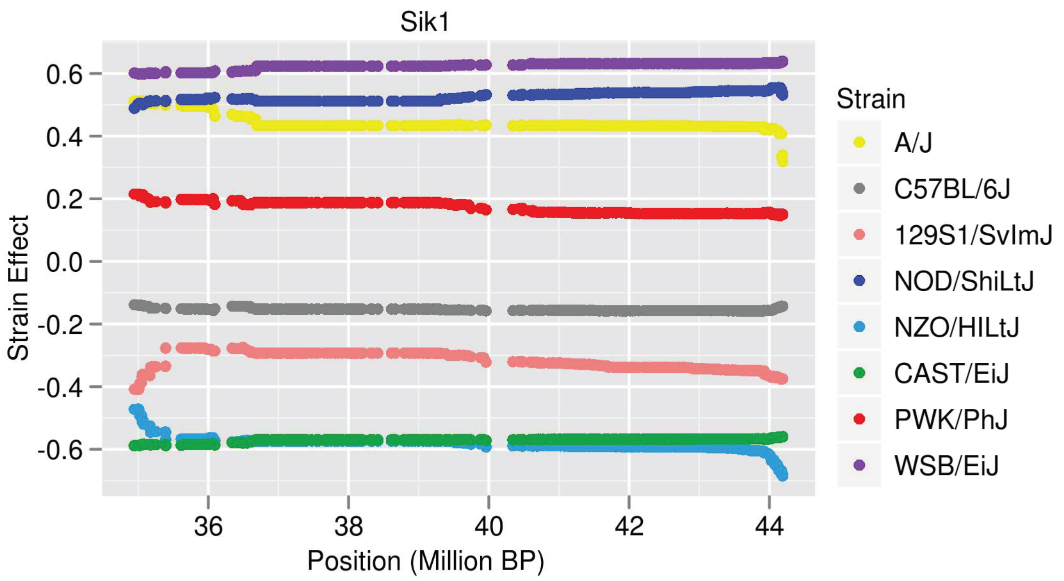

B.

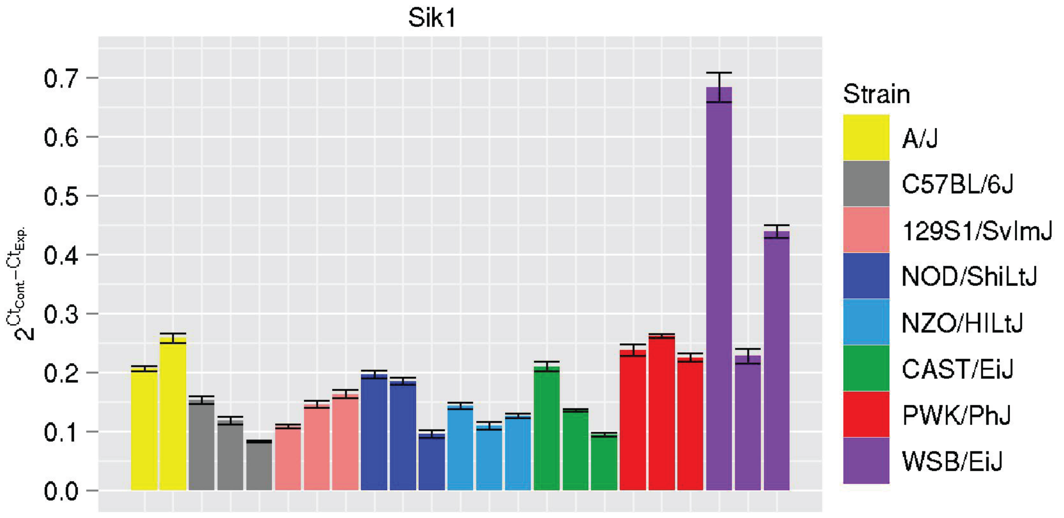

Figure S11

A.

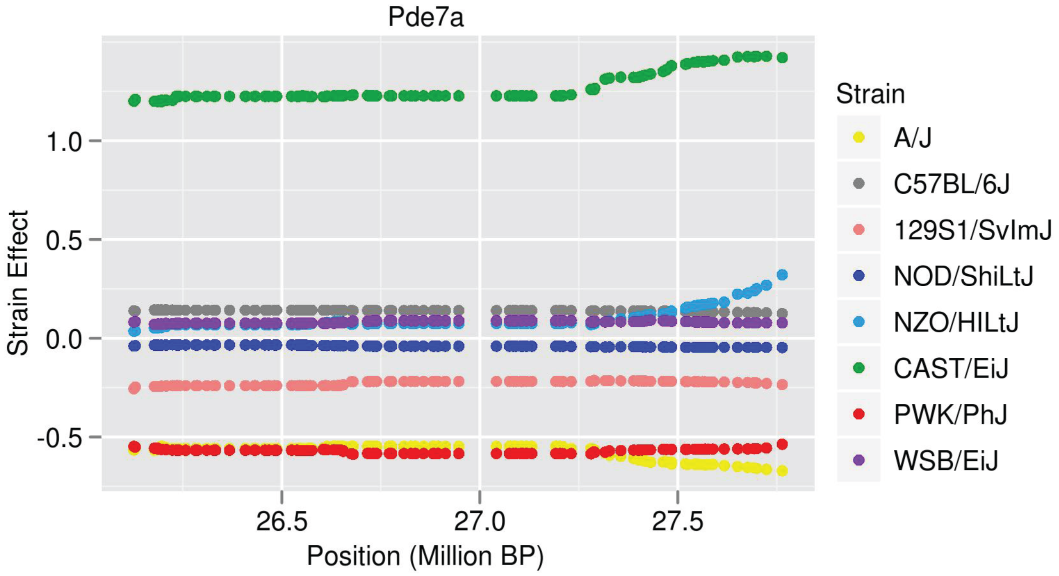

B.

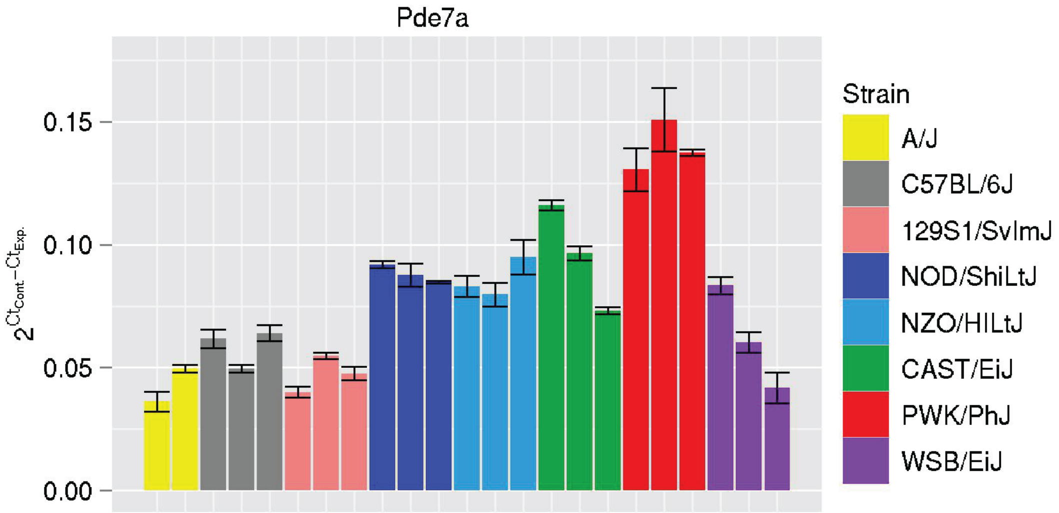

Figure S12

A.

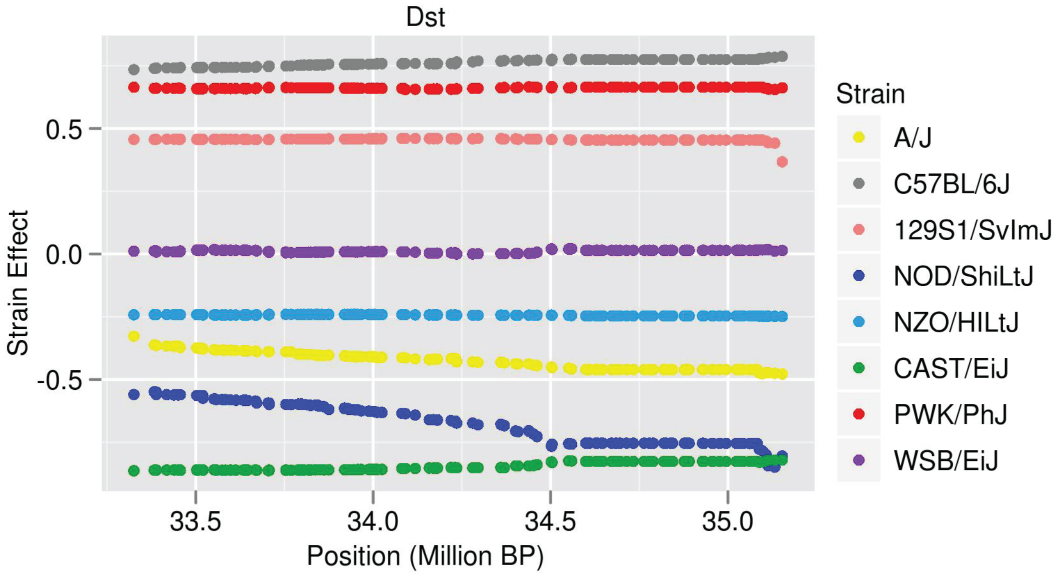

B.

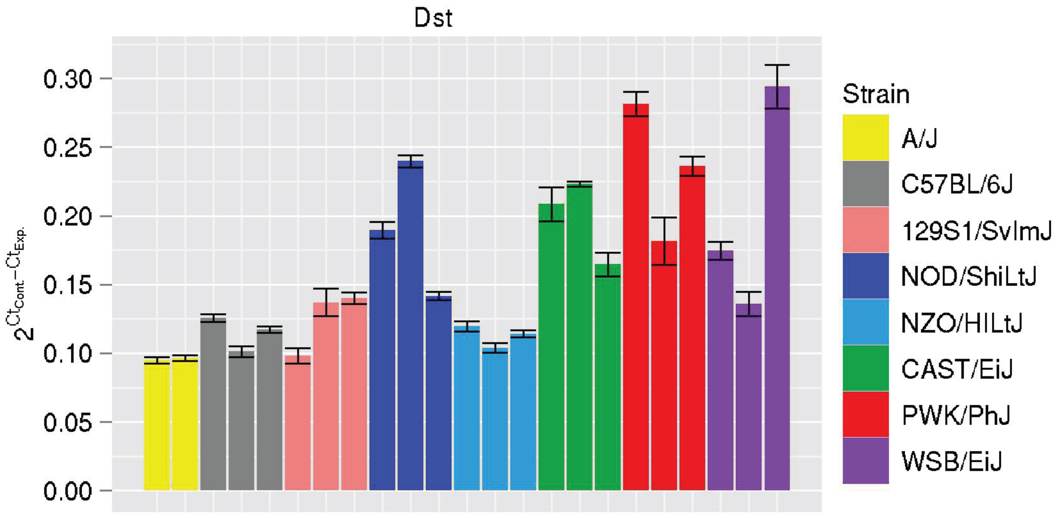

Figure S13

A.

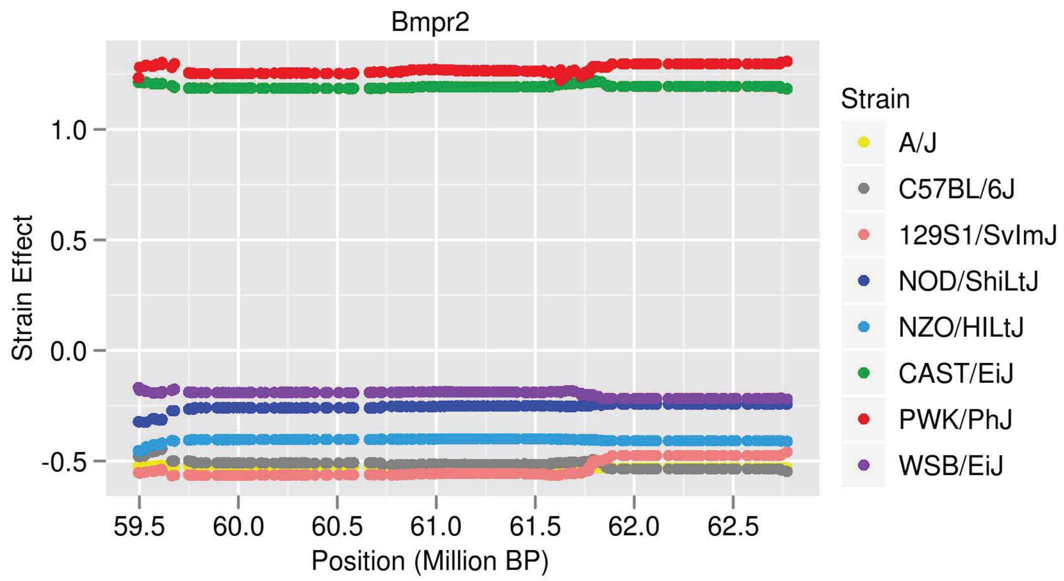

B.

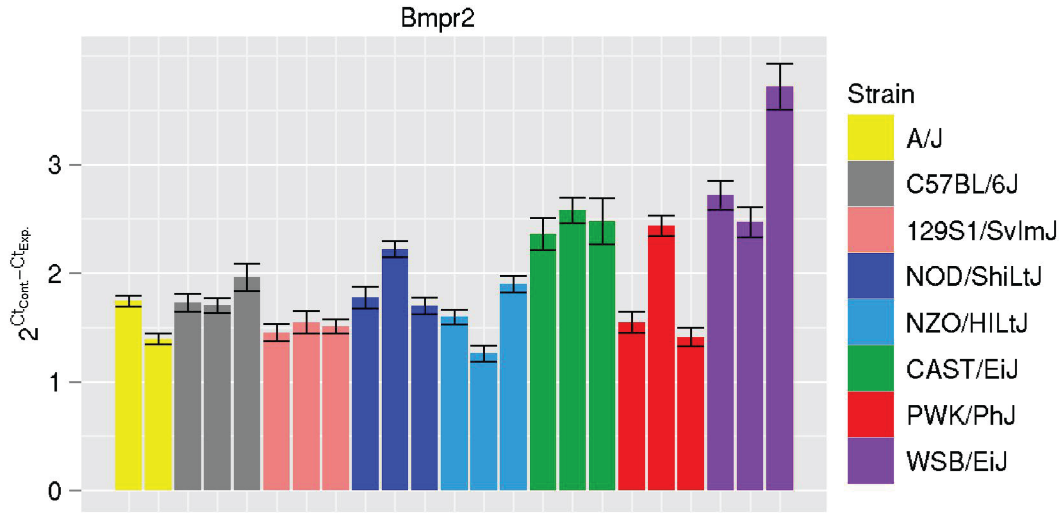

Figure S14

A.

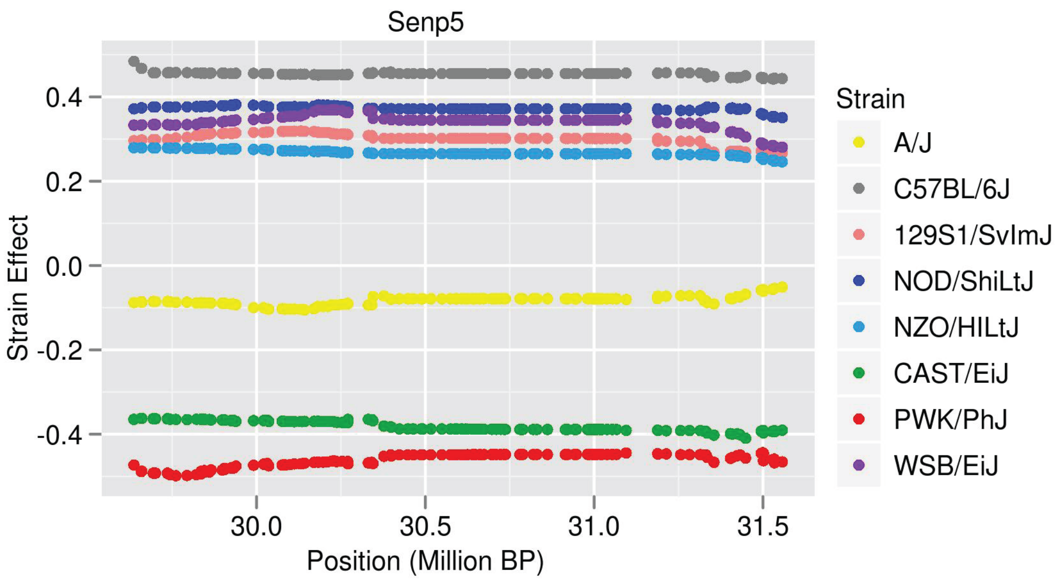

B.

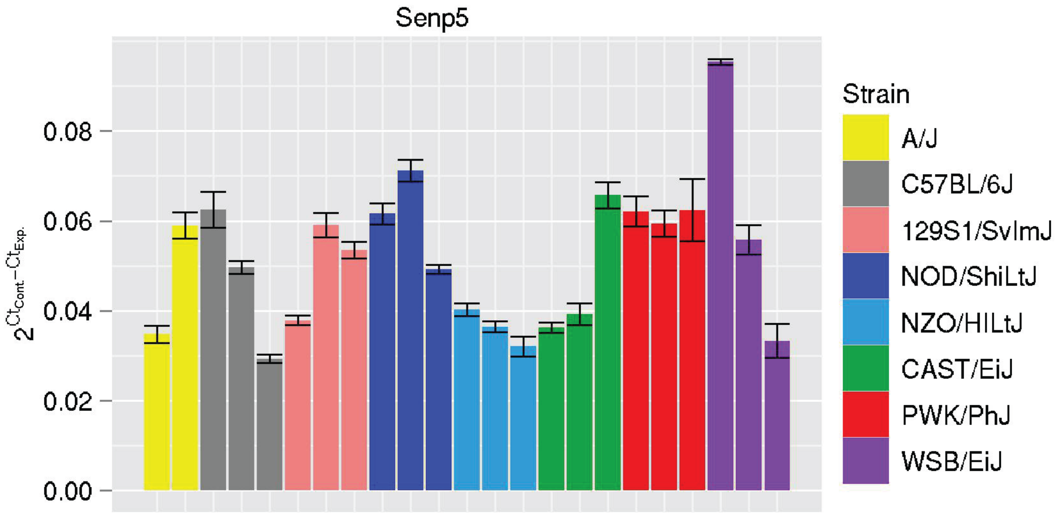

Figure S15

A.

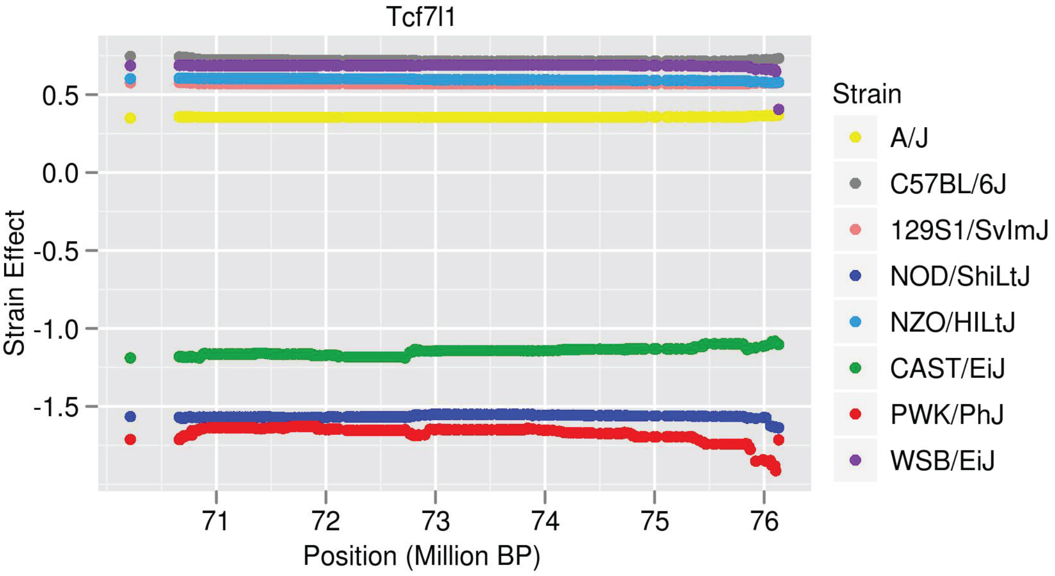

B.

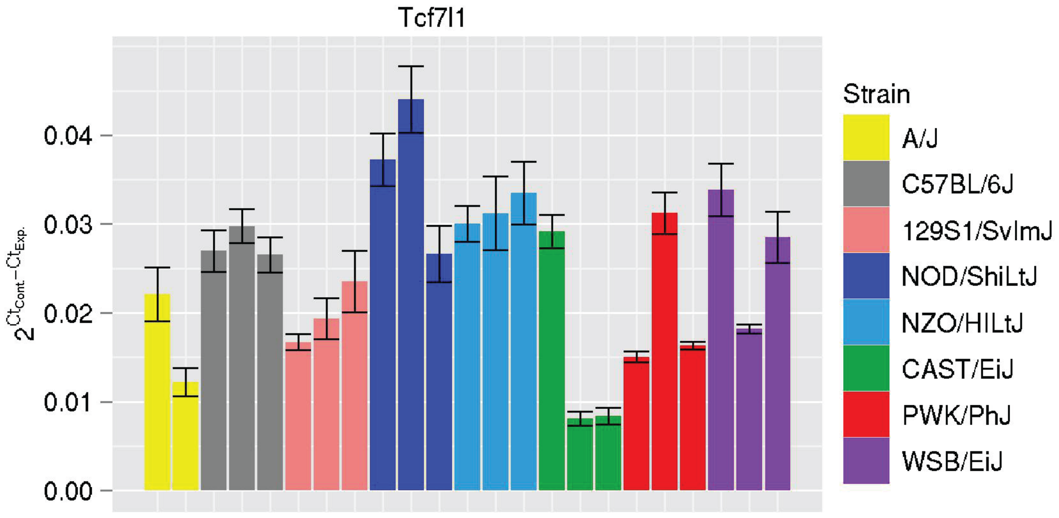

Figure S16

Figure S1-S16 Allele effect and qPCR plots. Allele effect and qPCR plots for the 7 remaining genes (Ifi27l2a is shown in Figure 2) with allele effects completely consistent with the qPCR data are shown in Figures S1-S7. Similarly the partially consistent genes are shown in Figures S8-S11 and those that failed to confirm are shown in Figures S12-16. All figures are divided into two parts: (A) Displays the allele effects (Aylor *et al*. 2011) for each marker in the 1.5 LOD drop support interval. This was done for each gene with a significant eQTL and is shown with the values colored by inbred founder strain. (B) Displays the barplots of the endogenous control corrected qPCR Ct data generated for each of the mice derived from the indicated inbred strain. The error bars indicate the standard error. The gene being regulated by the eQTL is indicated above both plots.

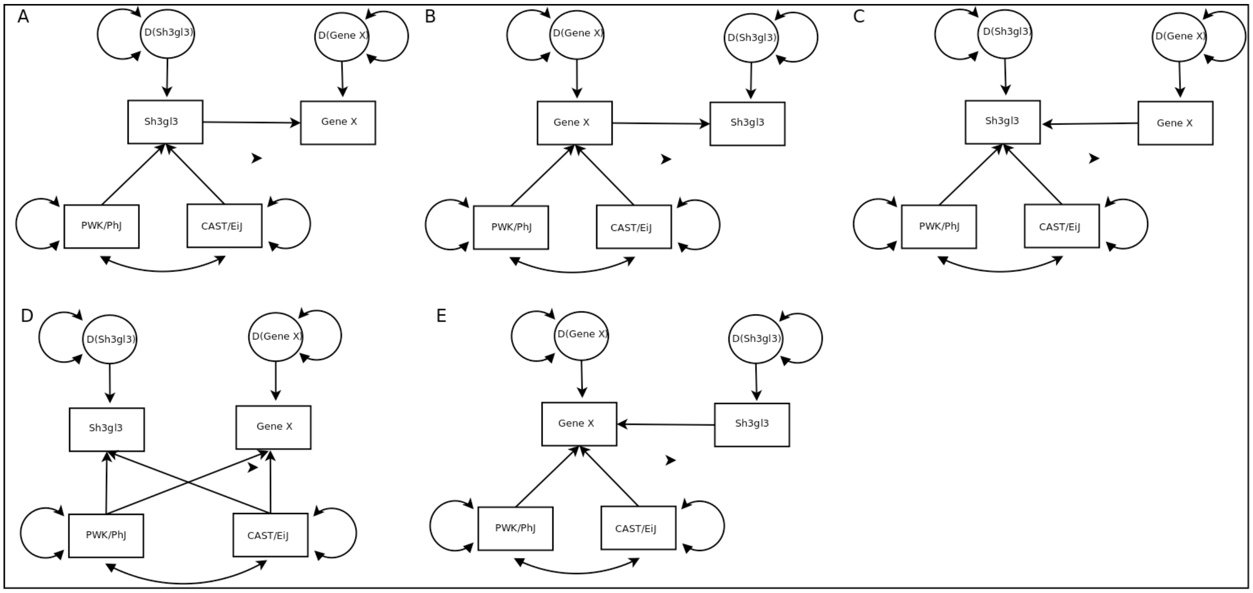

Figure S17 An example of the path model diagrams for the 5 specified structural equation models. Shown in RAM notation are the 5 path models for the *Sh3gl3* gene and its relevant allelic contributions from PWK/PhJ and CAST/EiJ which were selected by forward variable selection. Our interest was in comparing model A against B-E. Gene X in this case represents a given gene from all the genes in the LRI group. The boxes represent variables and the single-header arrows represent relationships (direct effects). The double headed arrows represent variance and covariance depending on whether they are pointed at themselves or other variables respectively. Circles are disturbances (also indicated by D(variable name)). Disturbances represent variance unexplained by the model.

Files S1-S9

Supporting Data

Files S1-S9 are available for download at http://www.g3journal.org/lookup/suppl/doi:10.1534/g3.111.001800/-/DC1.

Table S1 Enriched GO terms for up-regulated genes in each extreme phenotypic group

Both the Gene Ontology term (GO Term) and the Benjamini and Yekutieli (Benjamini and Yekutieli. 2001) false discovery rate (BY FDR) are shown for the genes that were up-regulated in the high and low response to infection groups. Only FDR values < .01 were kept here.

Table S1 is available for download as a csv file at http://www.g3journal.org/lookup/suppl/doi:10.1534/g3.111.001800/-/DC1.

Table S2 Strain contributions chosen by forward variable selection

Gene Name	Selected Strains	Allele effects
LOC675467	G,C,F,G	G,C
Ifi27l2a	G	G
Thnsl2	G,D,A,G	G,D
AK153595	G,D,F	G,D
BC022687	C,D,B,A	B,D,C
Sh3gl3	G,F	F,G
Clec16a	B,E,F	B
Kcmf1	F,H,B,D,G	F

Forward variable selection was used on the expected haplotype contributions to choose the most informative variables to include in the SEM models. The actual strain contributions chosen are shown in the “Selected Strains” column along with the smallest allele effect group in the “Allele effects” column. The letters correspond to strains in the following manner: A (A/J), B (C57BL/6J), C (129S1/SvImJ), D (NOD/ShiLtJ), E (NZO/HILtJ), F (CAST/EiJ), G (PWK/PhJ) and H (WSB/EiJ).

Table S3 Local SEM inferred causal relationships

Each of the eight validated genes (“From” column) were examined relative to all the other genes (“To” column) in their respective groups (HRI or LRI). The most significant marker interval was determined for each of the eight genes and the strain contributions for each was picked using forward variable selection. Five SEM models were fit comparing the possible relationships between the selected strains for a given marker interval and the genes (Aten *et al.* 2008). Several values were computed including the NB Score which is the model P-value (“Model P” column) divided by the next highest model P-value and log10 transformed. Other fit comparison measures are also shown, the RMSEA, SRMSR, CFI along with the Wald P-value. Only those gene relationships that had a score > 1, model P-value > .05, RMSEA score < .05, SRMSR < .1, CFI > .9 and Wald P-value < .05 were kept.

Table S3 is available for download as a csv file at http://www.g3journal.org/lookup/suppl/doi:10.1534/g3.111.001800/-/DC1.

Table S4 GO Categories for the genes reactive to Ifi27l2a , Sh3gl3 and Kcmf1

GO Term	P-value
A. Ifi27l2a	
transition metal ion transport	0.001
protein import into nucleus, translocation	0.002
iron ion transport	0.004
protein import into nucleus	0.006
regulation of endocytosis	0.006
positive regulation of endocytosis	0.006
regulation of phagocytosis	0.006
positive regulation of phagocytosis	0.006
nuclear import	0.006
metal ion transport	0.008
protein targeting	0.009
protein import	0.009
protein localization in nucleus	0.009
negative regulation of multicellular organismal process	0.009
cation transport	0.009
nucleocytoplasmic transport	0.012
nuclear transport	0.012
protein localization in organelle	0.015
regulation of vesicle-mediated transport	0.015
B. Sh3gl3	
multicellular organismal process	0
regulation of multicellular organismal process	0
mesoderm development	0.003
multicellular organismal development	0.004
cell migration	0.004
regulation of developmental process	0.004
negative regulation of cell growth	0.004
negative regulation of cell size	0.004
cellular component movement	0.005
patterning of blood vessels	0.006
olfactory bulb development	0.006
olfactory lobe development	0.006
retinal ganglion cell axon guidance	0.006
positive regulation of protein binding	0.006

regulation of protein binding	0.006
locomotion	0.007
anatomical structure formation involved in morphogenesis	0.007
establishment of protein localization	0.007
formation of primary germ layer	0.008
mesoderm formation	0.008
C. Kcmf1	
cholesterol metabolic process	0.007
sterol metabolic process	0.007
steroid metabolic process	0.010
response to radiation	0.020
arginine metabolic process	0.023
xenobiotic metabolic process	0.023
membrane budding	0.023
vesicle coating	0.023
striated muscle contraction	0.023
response to xenobiotic stimulus	0.023
coenzyme A metabolic process	0.023
dendrite development	0.023
cytidine to uridine editing	0.023
mRNA modification	0.023
protein sumoylation	0.023
regulation of protein sumoylation	0.023
positive regulation of protein sumoylation	0.023
nucleoside bisphosphate metabolic process	0.023
lipoprotein transport	0.023

The genes reactive to either *Ifi27l2a* (A) or *Sh3gl3* (B) or *Kcmf1* (C) were entered into a GO analysis focusing on overrepresentation of biological process terms. Shown are the top 20 GO terms and their P-values for all tables.

LITERATURE CITED

Aten, J., T. Fuller, A. Lusis and S. Horvath, 2008 Using genetic markers to orient the edges in quantitative trait networks: The NEO software. BMC Systems Biology **2:** 34.

Aylor, D. L., W. Valdar, W. Foulds-Mathes, R. J. Buus, R. A. Verdugo *et al*, 2011 Genetic analysis of complex traits in the emerging collaborative cross. Genome Research .

Benjamini, Y., and D. Yekutieli, 2001 The control of the false discovery rate in multiple testing under dependency. The Annals of Statistics **29:** 1165-1188.
