# Supplementary figures and images for: Expression Quantitative Trait Loci for Extreme Host Response to Influenza A in Pre-Collaborative Cross Mice
Source: G3 (Bethesda). 2012 Feb 1;2(2):213–21. doi: 10.1534/g3.111.001800 (PMC3284329; doi:10.1534/g3.111.001800)

A.

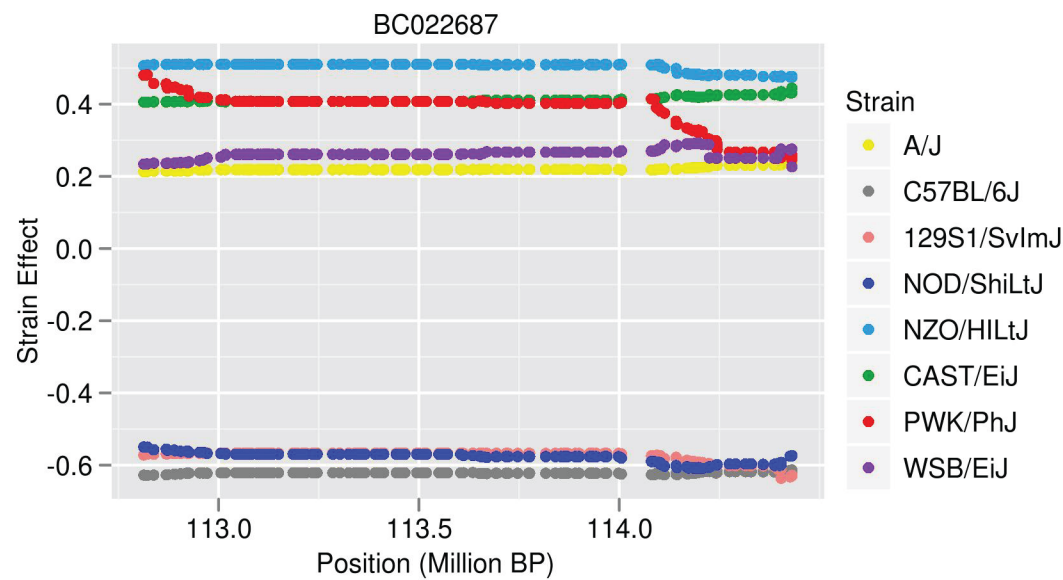

B.

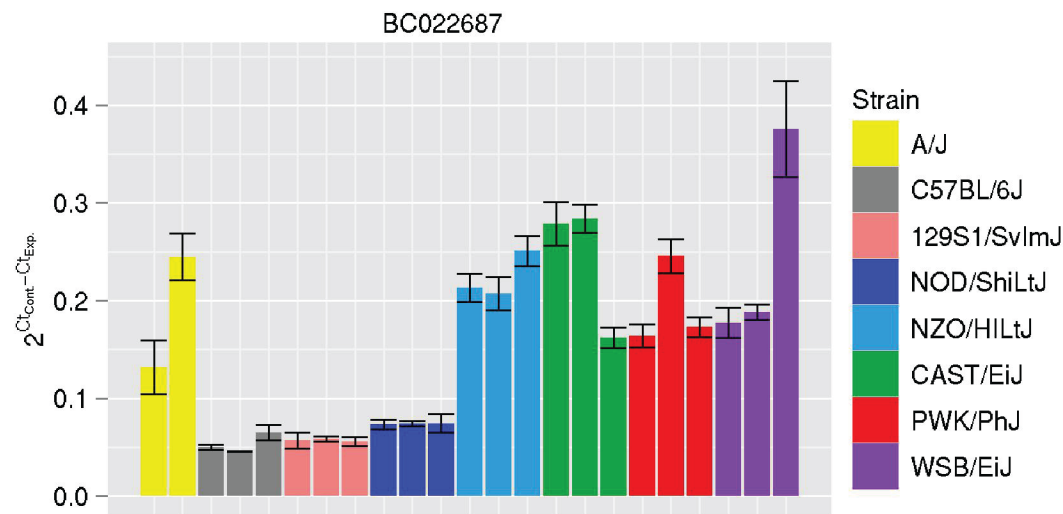

Figure S1

Supplement: Supporting Information [file supp_2.2.213_FigureS1.pdf]

A.

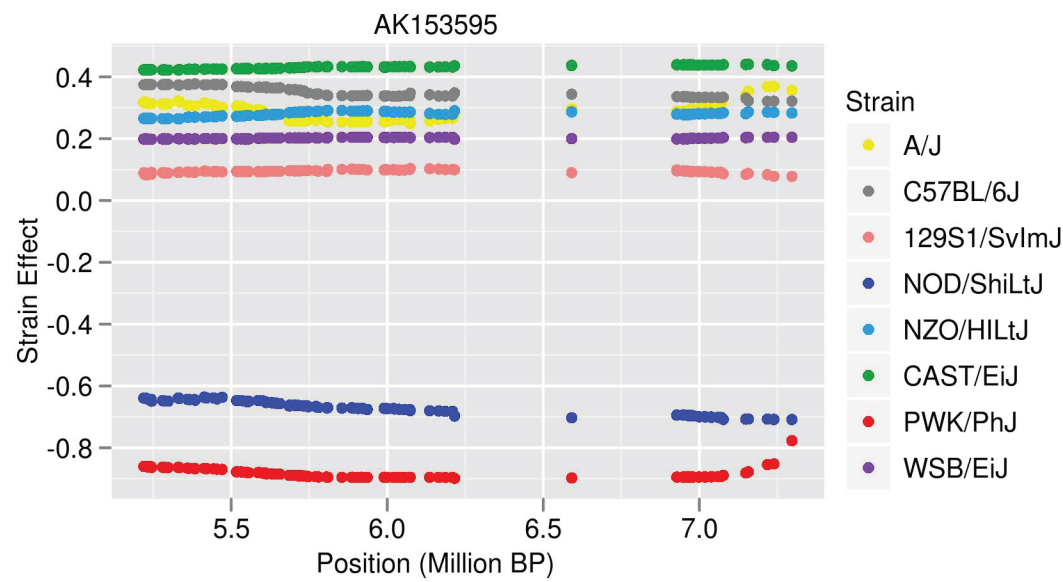

B.

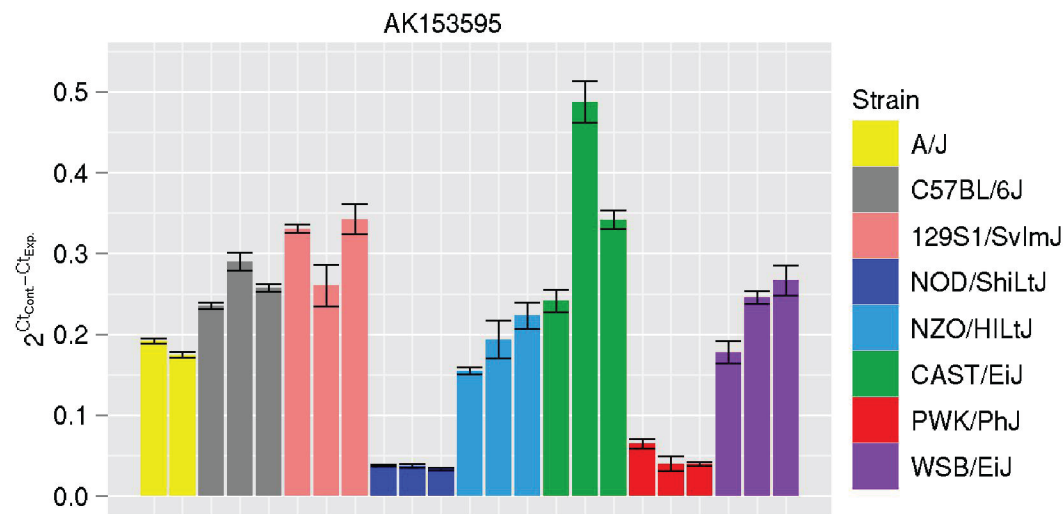

Figure S2

Supplement: Supporting Information [file supp_2.2.213_FigureS2.pdf]

A.

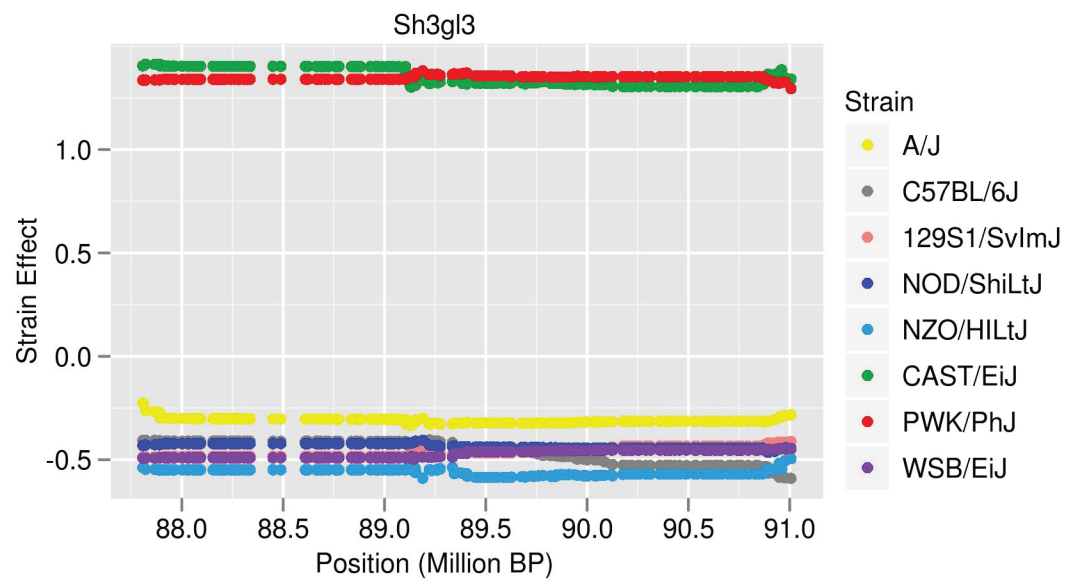

B.

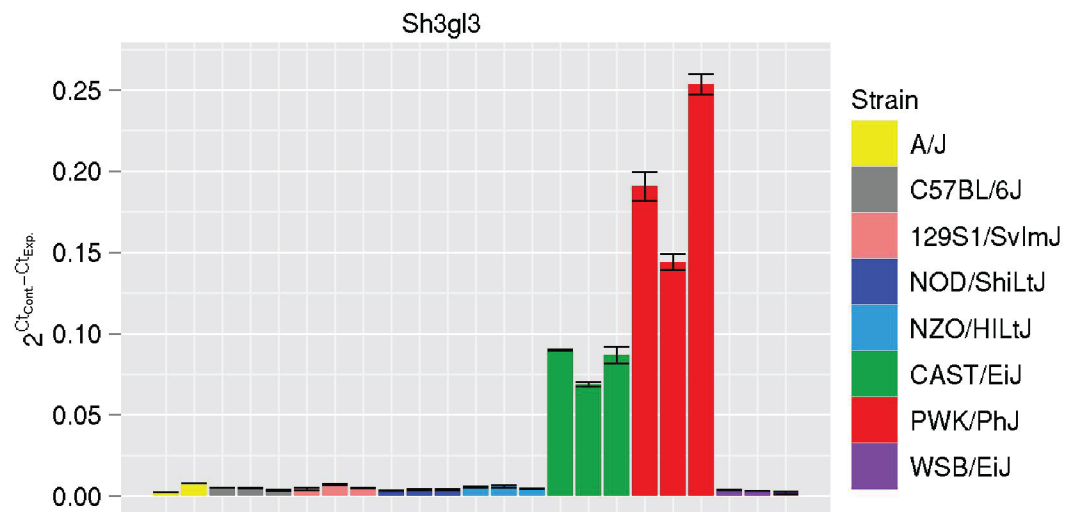

Figure S3

Supplement: Supporting Information [file supp_2.2.213_FigureS3.pdf]

A.

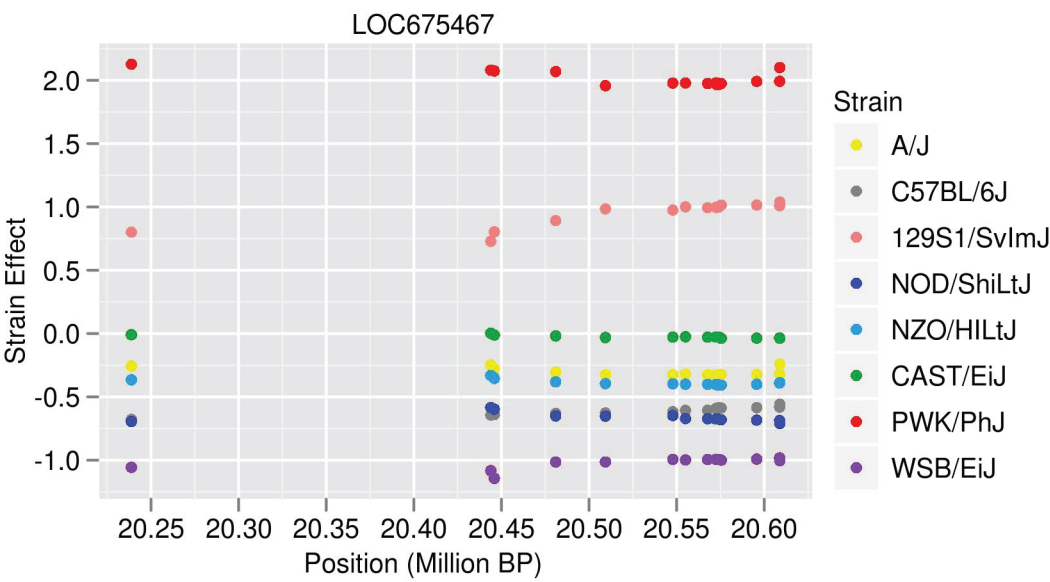

B.

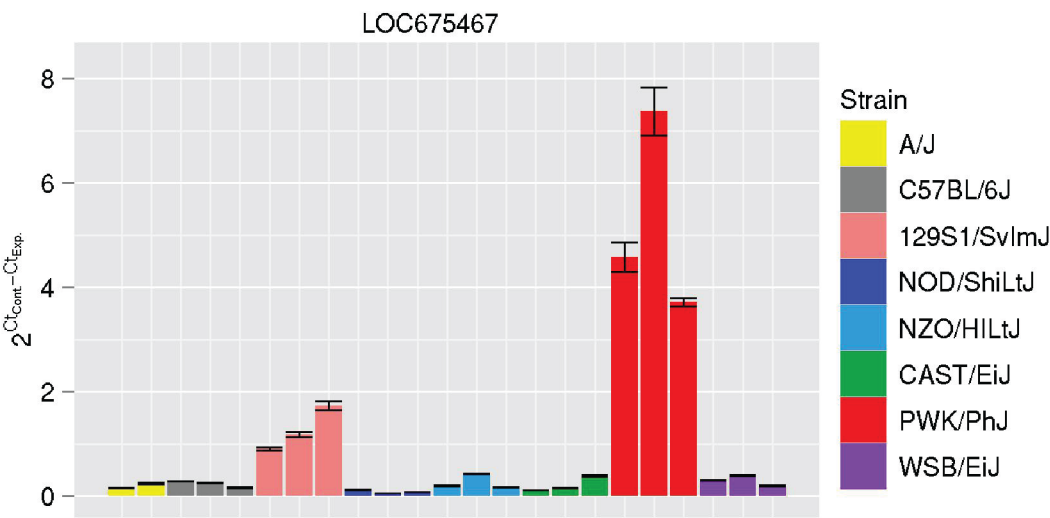

Figure S4

Supplement: Supporting Information [file supp_2.2.213_FigureS4.pdf]

A.

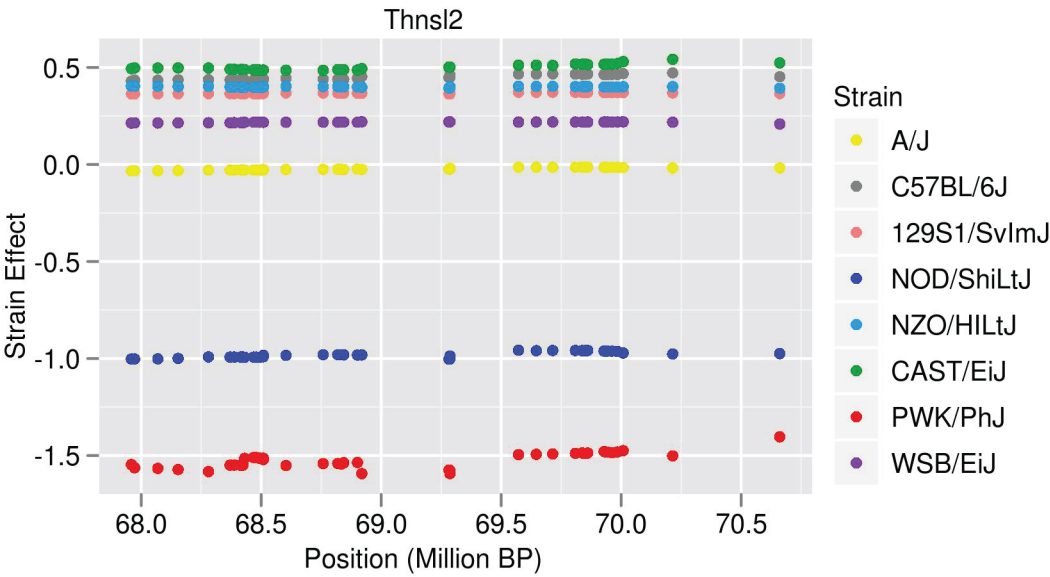

B.

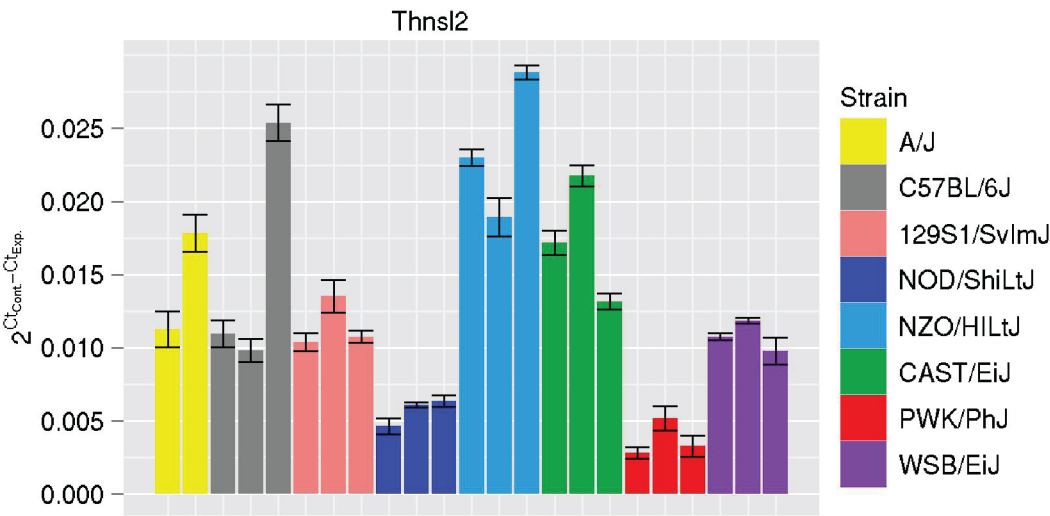

Figure S5

Supplement: Supporting Information [file supp_2.2.213_FigureS5.pdf]

A.

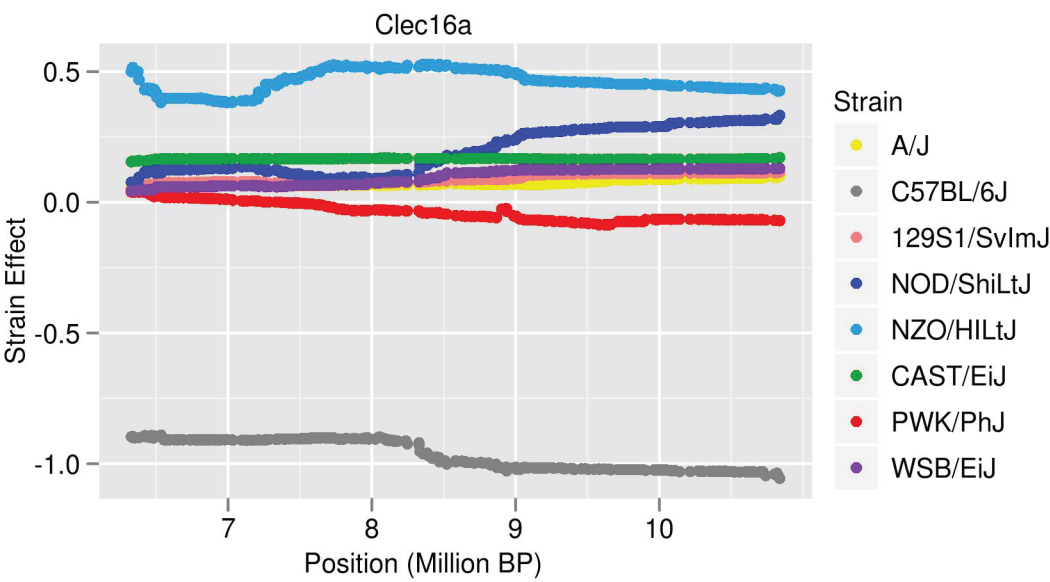

B.

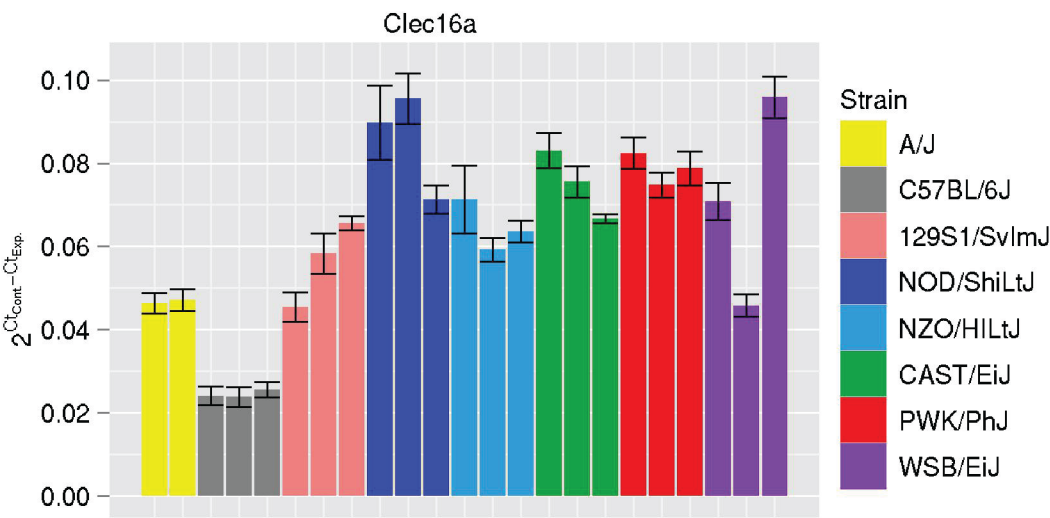

Figure S6

Supplement: Supporting Information [file supp_2.2.213_FigureS6.pdf]

A.

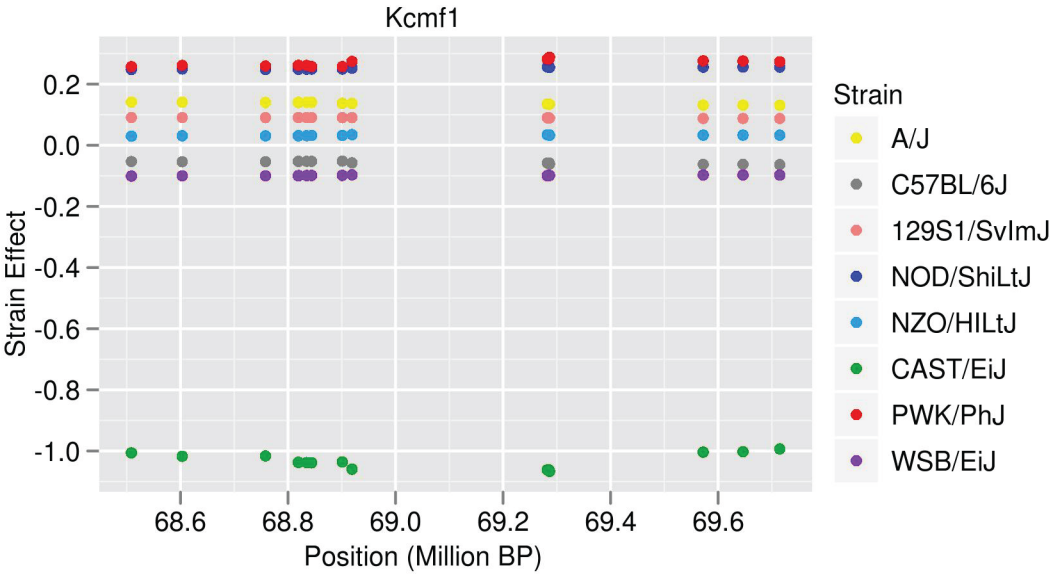

B.

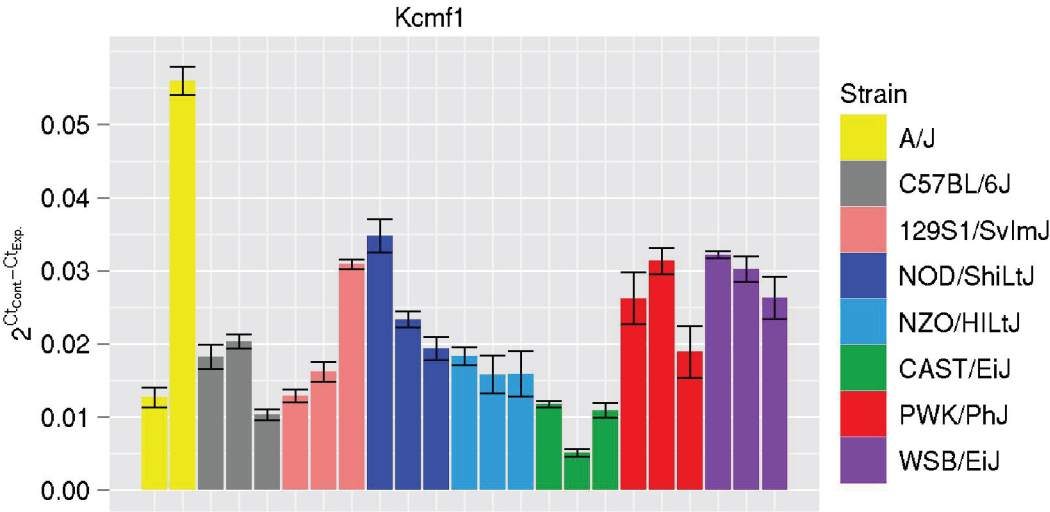

Figure S7

Supplement: Supporting Information [file supp_2.2.213_FigureS7.pdf]

A.

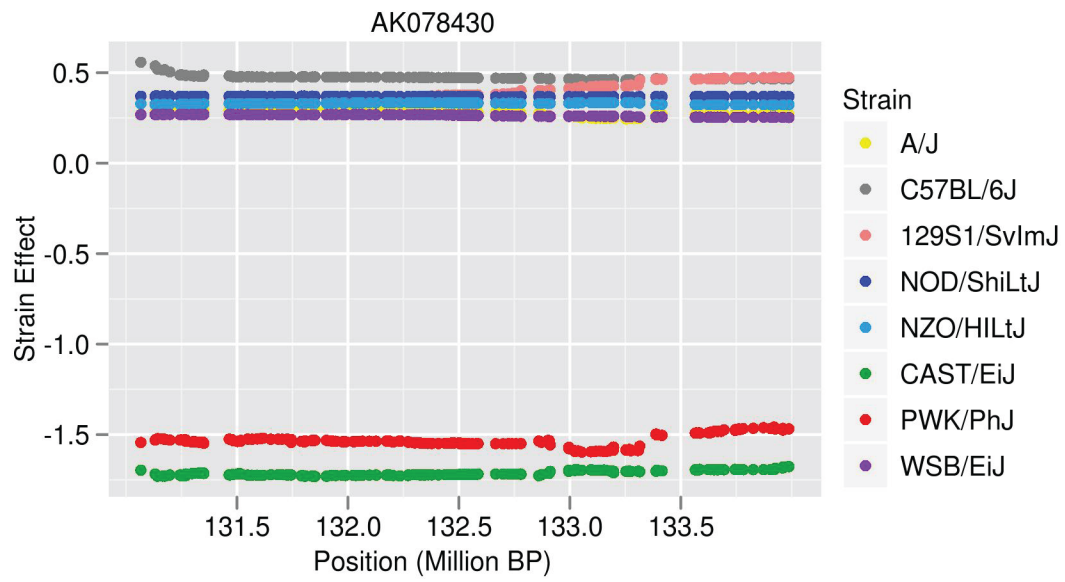

B.

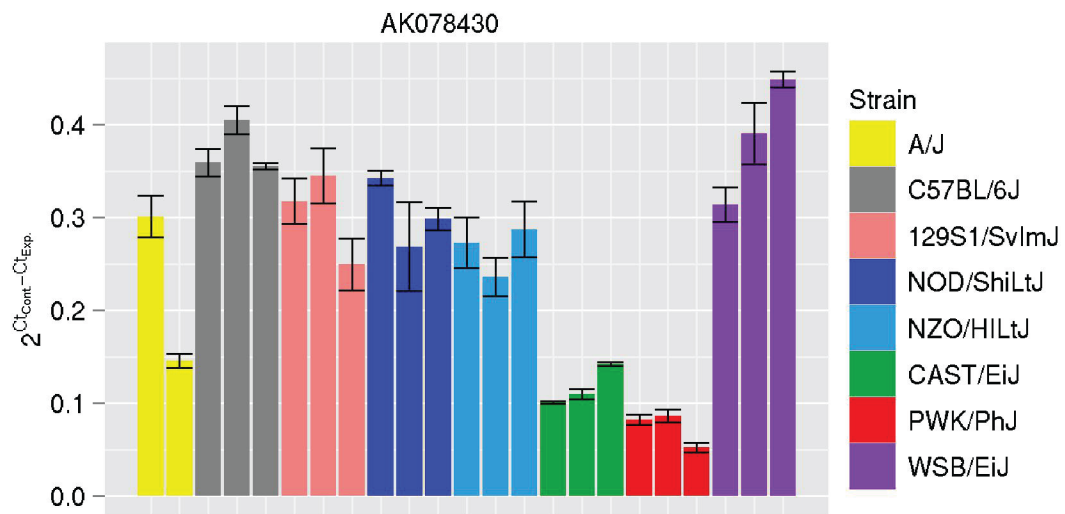

Figure S8

Supplement: Supporting Information [file supp_2.2.213_FigureS8.pdf]

A.

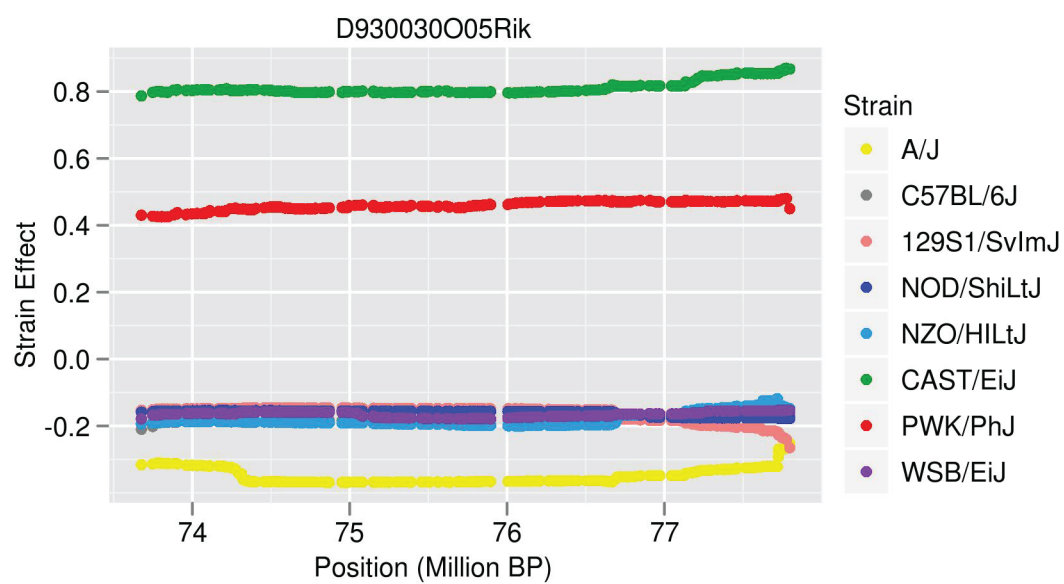

B.

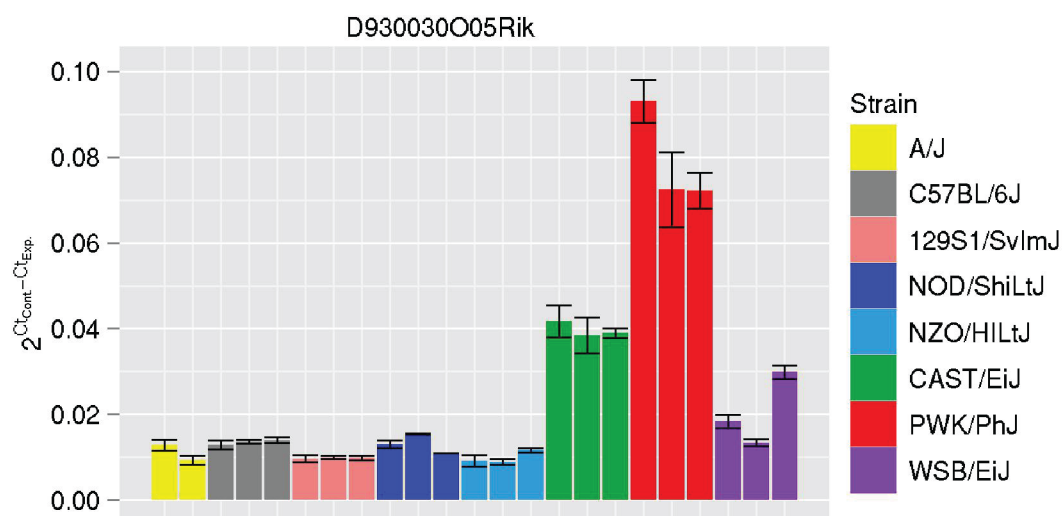

Figure S9

Supplement: Supporting Information [file supp_2.2.213_FigureS9.pdf]

A.

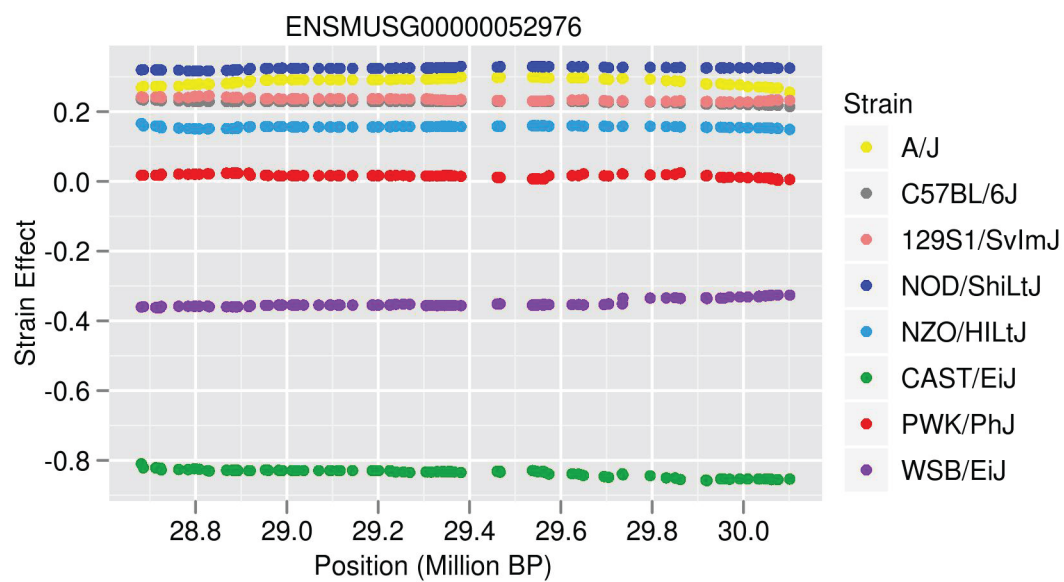

B.

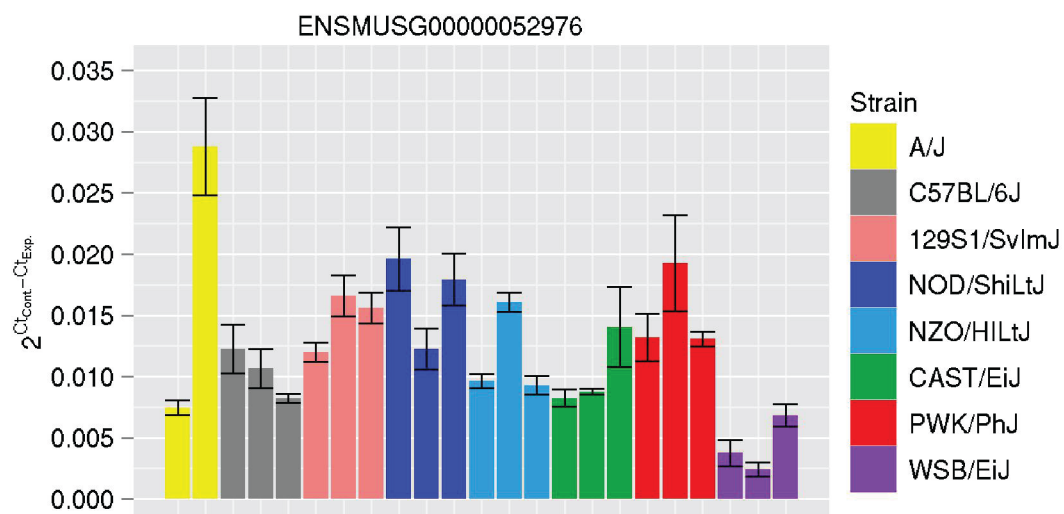

Figure S10

Supplement: Supporting Information [file supp_2.2.213_FigureS10.pdf]

A.

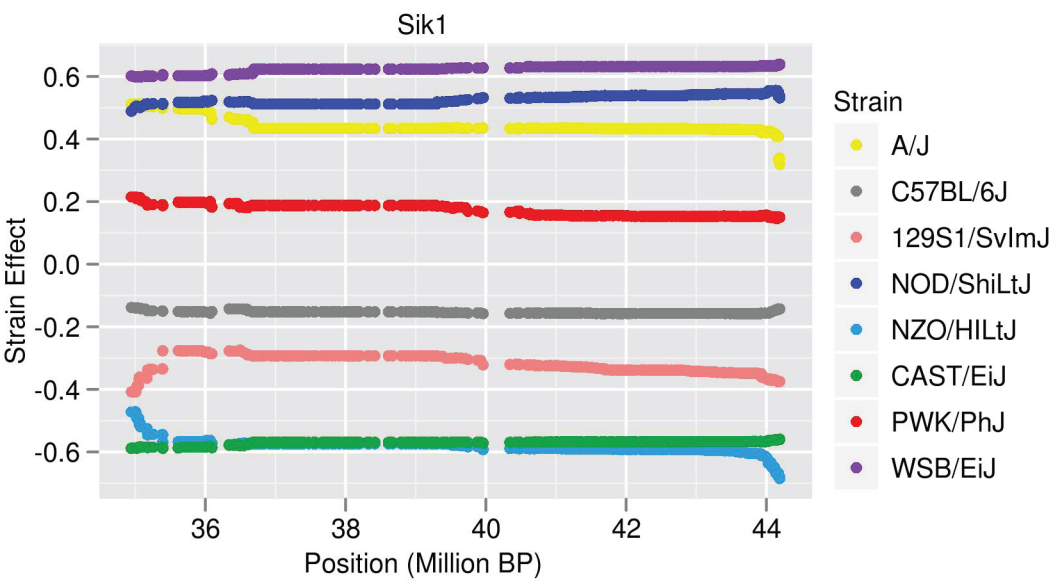

B.

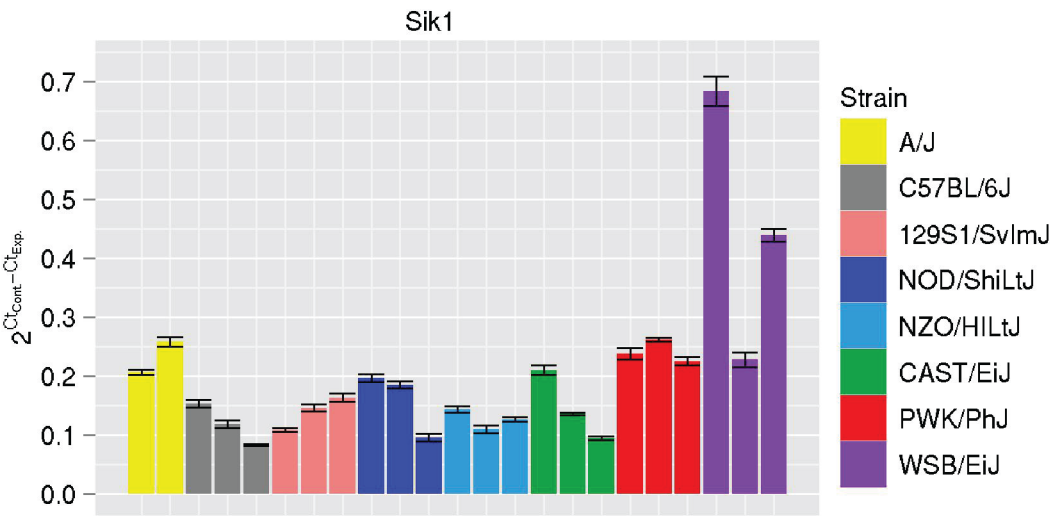

Figure S11

Supplement: Supporting Information [file supp_2.2.213_FigureS11.pdf]

A.

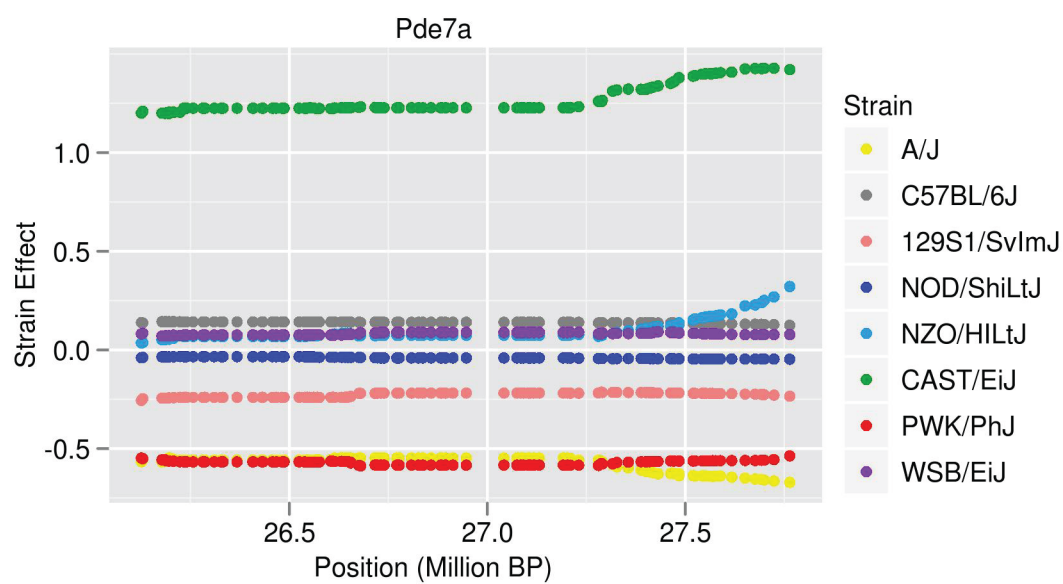

B.

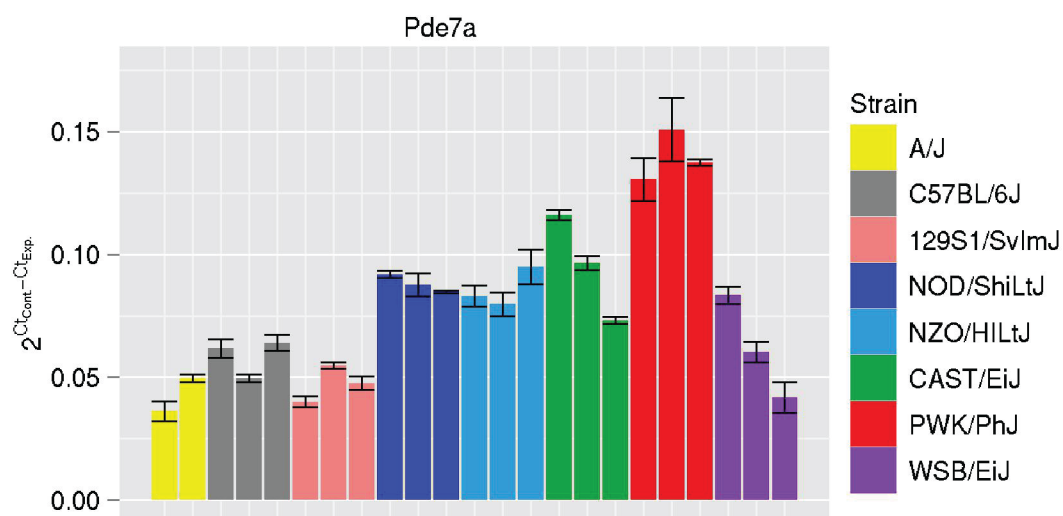

Figure S12

Supplement: Supporting Information [file supp_2.2.213_FigureS12.pdf]

A.

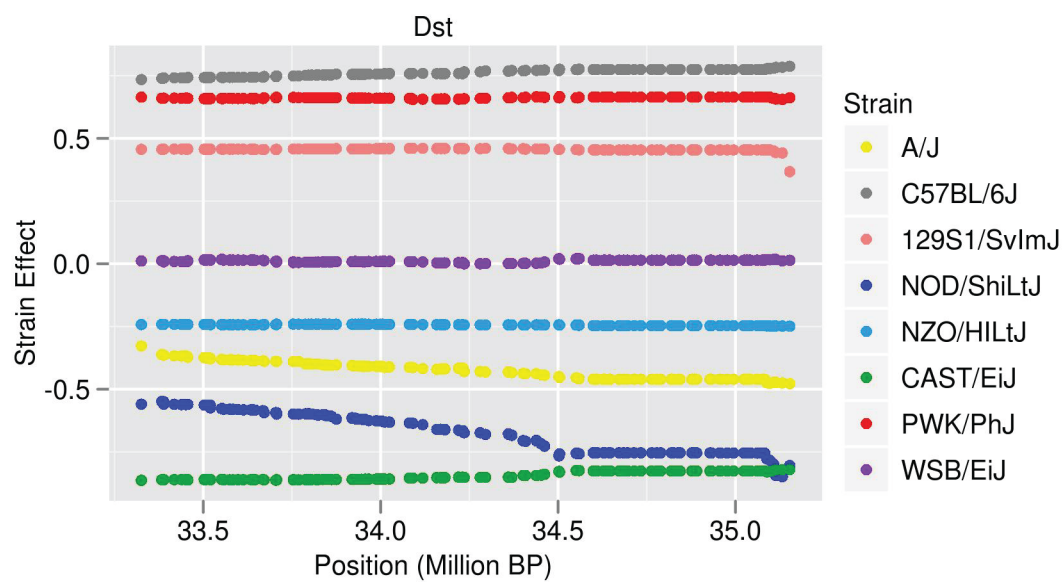

B.

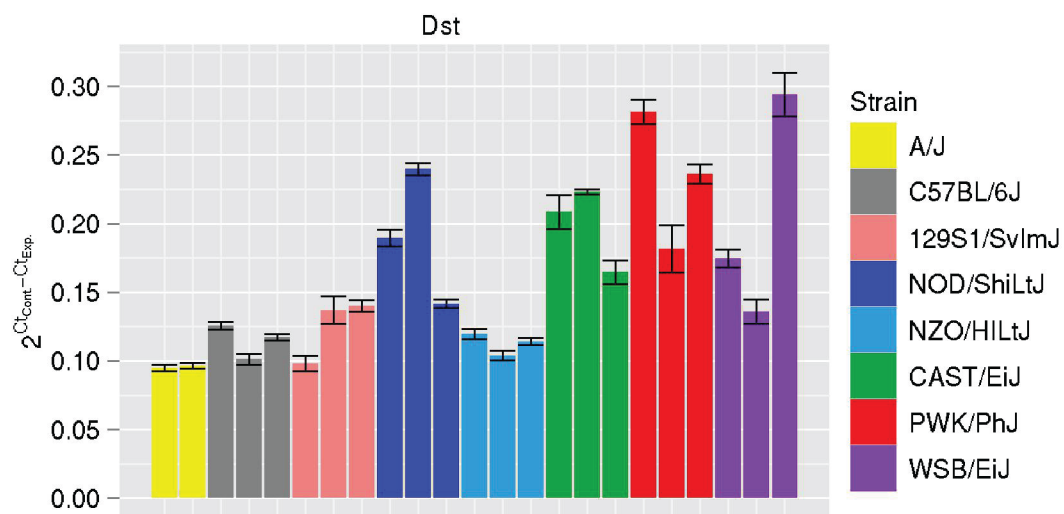

Figure S13

Supplement: Supporting Information [file supp_2.2.213_FigureS13.pdf]

A.

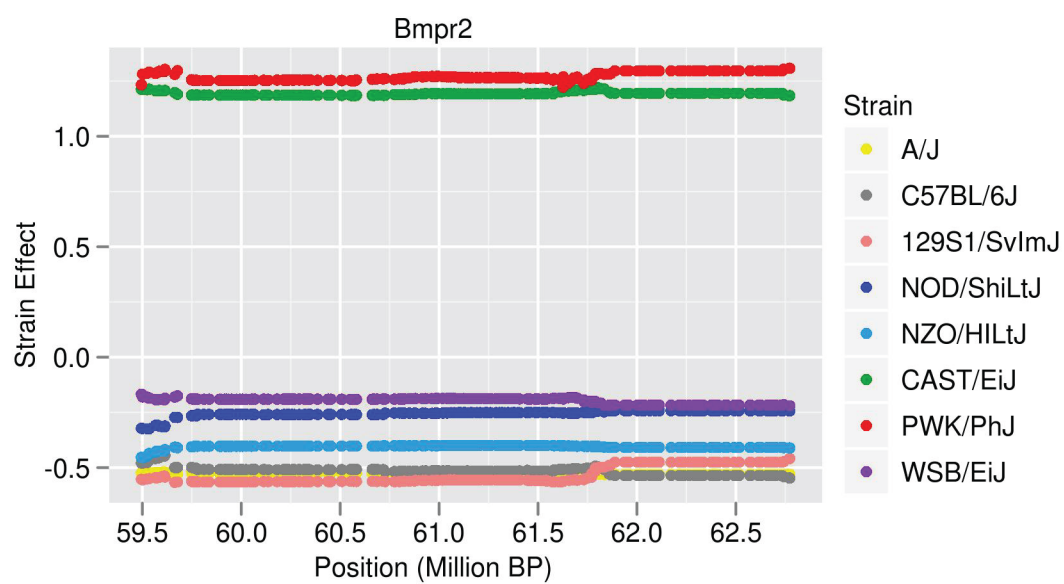

B.

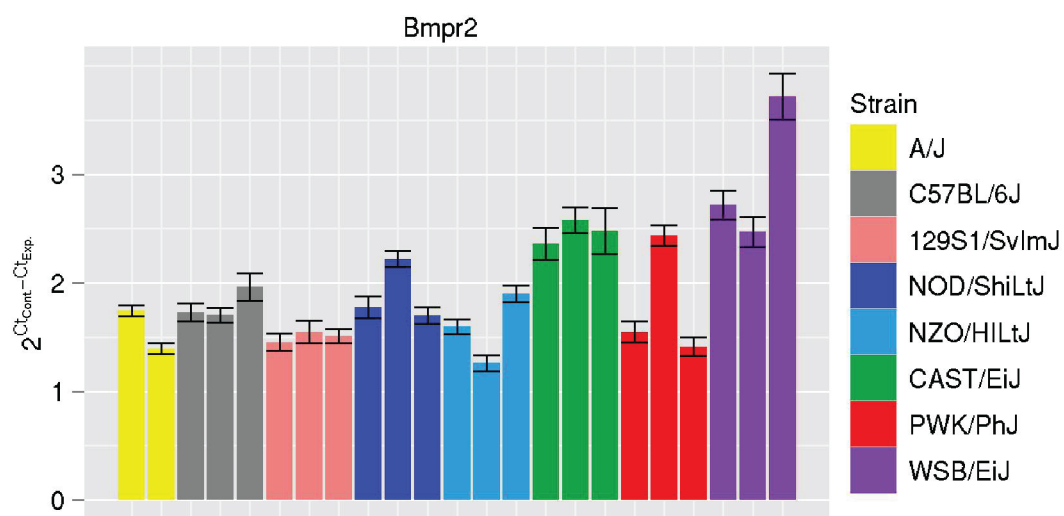

Figure S14

Supplement: Supporting Information [file supp_2.2.213_FigureS14.pdf]

A.

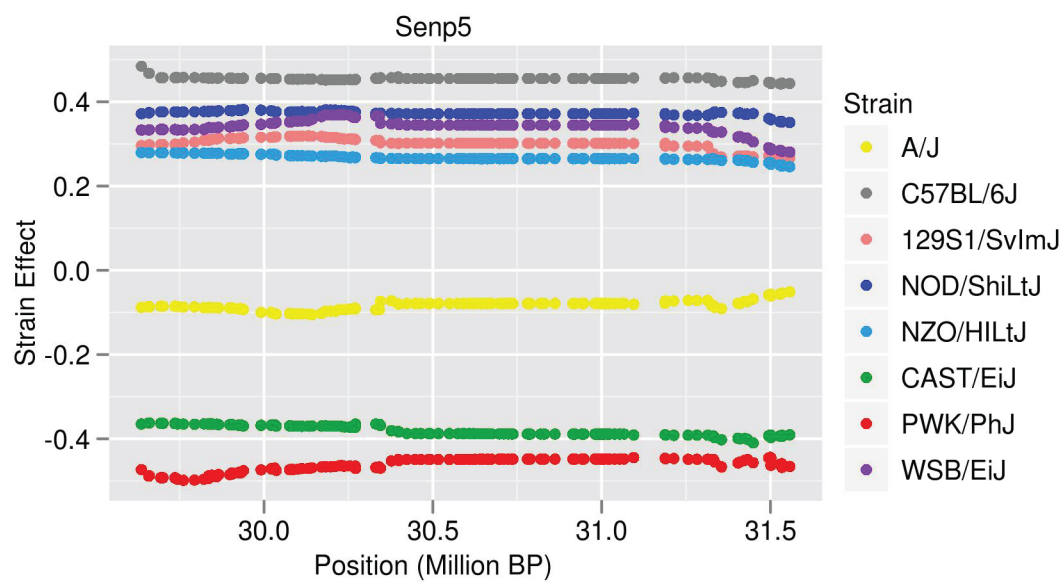

B.

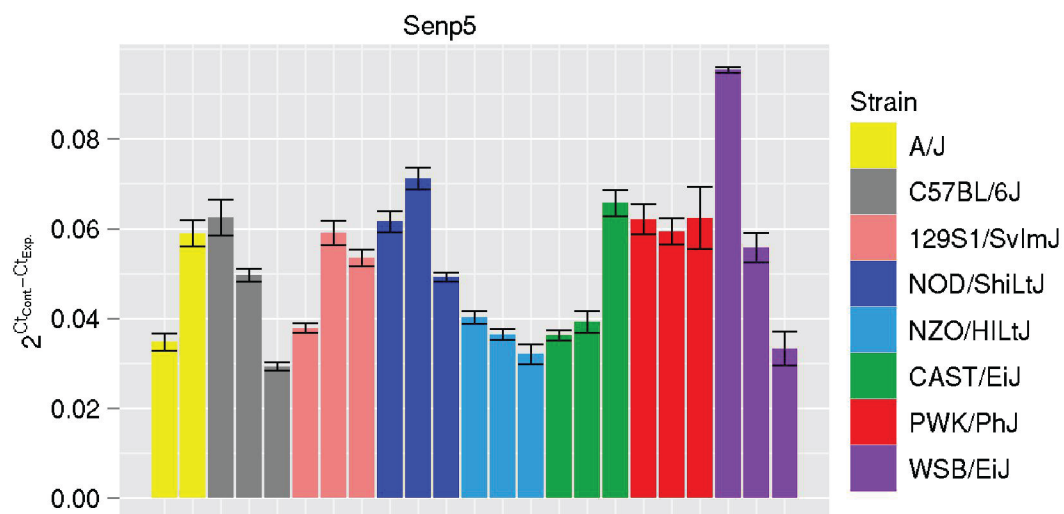

Figure S15

Supplement: Supporting Information [file supp_2.2.213_FigureS15.pdf]
