# Supplementary material for: Expression Quantitative Trait Loci for Extreme Host Response to Influenza A in Pre-Collaborative Cross Mice
Source: G3 (Bethesda). 2012 Feb 1;2(2):213–21. doi: 10.1534/g3.111.001800 (PMC3284329; doi:10.1534/g3.111.001800)
Supplement: Supporting Information [file supp_2.2.213_FigureS16.pdf]

A.

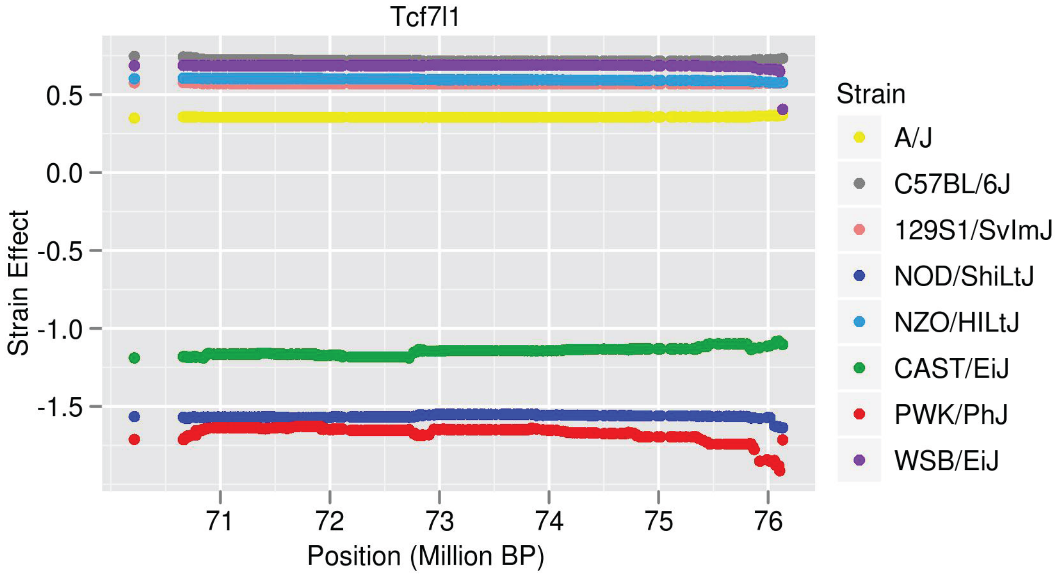

B.

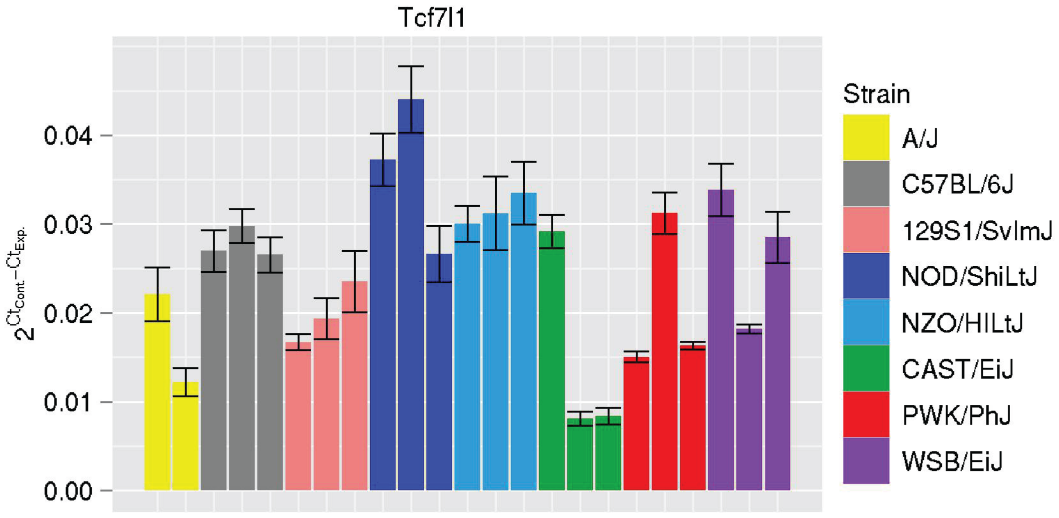

Figure S16

Figure S1-S16 Allele effect and qPCR plots. Allele effect and qPCR plots for the 7 remaining genes (Ifi27l2a is shown in Figure 2) with allele effects completely consistent with the qPCR data are shown in Figures S1-S7. Similarly the partially consistent genes are shown in Figures S8-S11 and those that failed to confirm are shown in Figures S12-16. All figures are divided into two parts: (A) Displays the allele effects (Aylor *et al*. 2011) for each marker in the 1.5 LOD drop support interval. This was done for each gene with a significant eQTL and is shown with the values colored by inbred founder strain. (B) Displays the barplots of the endogenous control corrected qPCR Ct data generated for each of the mice derived from the indicated inbred strain. The error bars indicate the standard error. The gene being regulated by the eQTL is indicated above both plots.
