# Supplementary material for: Expression Quantitative Trait Loci for Extreme Host Response to Influenza A in Pre-Collaborative Cross Mice
Source: G3 (Bethesda). 2012 Feb 1;2(2):213–21. doi: 10.1534/g3.111.001800 (PMC3284329; doi:10.1534/g3.111.001800)
Supplement: Supporting Information [file supp_2.2.213_FigureS17.pdf]

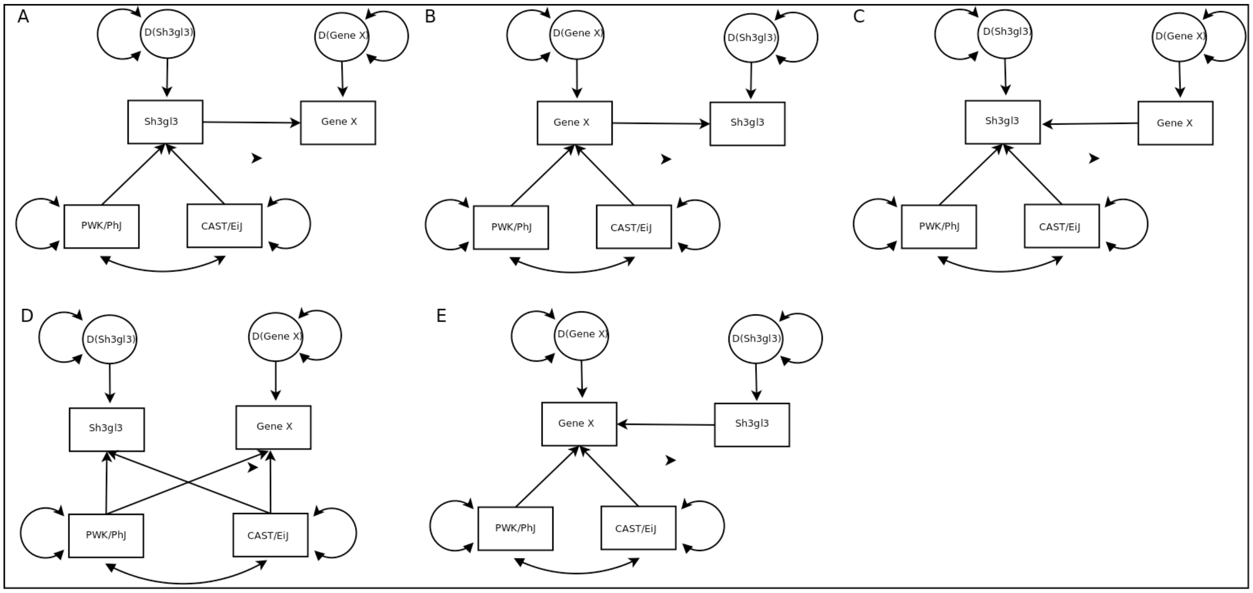

Figure S17 An example of the path model diagrams for the 5 specified structural equation models. Shown in RAM notation are the 5 path models for the *Sh3gl3* gene and its relevant allelic contributions from PWK/PhJ and CAST/EiJ which were selected by forward variable selection. Our interest was in comparing model A against B-E. Gene X in this case represents a given gene from all the genes in the LRI group. The boxes represent variables and the single-header arrows represent relationships (direct effects). The double headed arrows represent variance and covariance depending on whether they are pointed at themselves or other variables respectively. Circles are disturbances (also indicated by D(variable name)). Disturbances represent variance unexplained by the model.
