# Supplementary material for: Expression Quantitative Trait Loci for Extreme Host Response to Influenza A in Pre-Collaborative Cross Mice
Source: G3 (Bethesda). 2012 Feb 1;2(2):213–21. doi: 10.1534/g3.111.001800 (PMC3284329; doi:10.1534/g3.111.001800)
Supplement: Supporting Information [file supp_2.2.213_TableS2.pdf]

Table S2 Strain contributions chosen by forward variable selection

Gene Name	Selected Strains	Allele effects
LOC675467	G,C,F,G	G,C
Ifi27l2a	G	G
Thnsl2	G,D,A,G	G,D
AK153595	G,D,F	G,D
BC022687	C,D,B,A	B,D,C
Sh3gl3	G,F	F,G
Clec16a	B,E,F	B
Kcmf1	F,H,B,D,G	F

Forward variable selection was used on the expected haplotype contributions to choose the most informative variables to include in the SEM models. The actual strain contributions chosen are shown in the "Selected Strains" column along with the smallest allele effect group in the "Allele effects" column. The letters correspond to strains in the following manner: A (A/J), B (C57BL/6J), C (129S1/SvImJ), D (NOD/ShiLtJ), E (NZO/HILtJ), F (CAST/EiJ), G (PWK/PhJ) and H (WSB/EiJ).
