# Supplementary material for: Expression Quantitative Trait Loci for Extreme Host Response to Influenza A in Pre-Collaborative Cross Mice
Source: G3 (Bethesda). 2012 Feb 1;2(2):213–21. doi: 10.1534/g3.111.001800 (PMC3284329; doi:10.1534/g3.111.001800)
Supplement: Supporting Information [file supp_2.2.213_TableS4.pdf]

Table S4 GO Categories for the genes reactive to Ifi27l2a , Sh3gl3 and Kcmf1

GO Term	P-value
A. Ifi27l2a	
transition metal ion transport	0.001
protein import into nucleus, translocation	0.002
iron ion transport	0.004
protein import into nucleus	0.006
regulation of endocytosis	0.006
positive regulation of endocytosis	0.006
regulation of phagocytosis	0.006
positive regulation of phagocytosis	0.006
nuclear import	0.006
metal ion transport	0.008
protein targeting	0.009
protein import	0.009
protein localization in nucleus	0.009
negative regulation of multicellular organismal process	0.009
cation transport	0.009
nucleocytoplasmic transport	0.012
nuclear transport	0.012
protein localization in organelle	0.015
regulation of vesicle-mediated transport	0.015
B. Sh3gl3	
multicellular organismal process	0
regulation of multicellular organismal process	0
mesoderm development	0.003
multicellular organismal development	0.004
cell migration	0.004
regulation of developmental process	0.004
negative regulation of cell growth	0.004
negative regulation of cell size	0.004
cellular component movement	0.005
patterning of blood vessels	0.006
olfactory bulb development	0.006
olfactory lobe development	0.006
retinal ganglion cell axon guidance	0.006
positive regulation of protein binding	0.006

regulation of protein binding	0.006
locomotion	0.007
anatomical structure formation involved in morphogenesis	0.007
establishment of protein localization	0.007
formation of primary germ layer	0.008
mesoderm formation	0.008
C. Kcmf1	
cholesterol metabolic process	0.007
sterol metabolic process	0.007
steroid metabolic process	0.010
response to radiation	0.020
arginine metabolic process	0.023
xenobiotic metabolic process	0.023
membrane budding	0.023
vesicle coating	0.023
striated muscle contraction	0.023
response to xenobiotic stimulus	0.023
coenzyme A metabolic process	0.023
dendrite development	0.023
cytidine to uridine editing	0.023
mRNA modification	0.023
protein sumoylation	0.023
regulation of protein sumoylation	0.023
positive regulation of protein sumoylation	0.023
nucleoside bisphosphate metabolic process	0.023
lipoprotein transport	0.023

The genes reactive to either *Ifi27l2a* (A) or *Sh3gl3* (B) or *Kcmf1* (C) were entered into a GO analysis focusing on overrepresentation of biological process terms. Shown are the top 20 GO terms and their P-values for all tables.
